# Fibrosis in Immune-Mediated and Autoimmune Disorders

**DOI:** 10.3390/jcm14186636

**Published:** 2025-09-20

**Authors:** Magdalena Żurawek, Iwona Ziółkowska-Suchanek, Katarzyna Iżykowska

**Affiliations:** Institute of Human Genetics, Polish Academy of Sciences, 60-479 Poznan, Poland; magdalena.zurawek@igcz.poznan.pl (M.Ż.); iwona.ziolkowska@igcz.poznan.pl (I.Z.-S.)

**Keywords:** fibrosis, autoimmune disorders, targeted therapies

## Abstract

Fibrosis is a pathological process characterized by the excessive accumulation of extracellular matrix (ECM), particularly collagen, leading to tissue scarring, architectural distortion, and organ dysfunction. While fibrosis is a physiological component of wound healing, its persistence and dysregulation can drive chronic tissue damage and organ dysfunction. In autoimmune diseases, fibrosis arises from prolonged inflammation and immune system dysregulation, creating a vicious cycle that exacerbates tissue injury and promotes disease progression. This review provides a comprehensive overview of the fibrotic processes across a range of immune-mediated and autoimmune conditions, including systemic sclerosis (SSc), morphea, autoimmune hepatitis (AIH), systemic lupus erythematosus (SLE), Sjögren’s syndrome (SS), inflammatory bowel disease (IBD), and rheumatoid arthritis (RA), Finally, we discuss current and emerging antifibrotic strategies aimed at interrupting pathological ECM remodeling and restoring tissue homeostasis.

## 1. Introduction

Fibrosis is a process characterized by the excessive accumulation of extracellular matrix components, particularly collagen, leading to the thickening and stiffening of tissues and organs [1,2]. It is a hallmark of many chronic diseases. In autoimmune disorders, fibrosis represents a critical mechanism driving disease progression and, unfortunately, emerges as a significant complication that contributes to organ dysfunction, morbidity, and, in some cases, mortality. This review explores the role of fibrosis in autoimmune diseases, delving into its underlying mechanisms, impact on various organs, and potential therapeutic strategies.

### 1.1. “Good” Fibrosis—A Physiological Process in Tissue Repair

**Figure 1:**
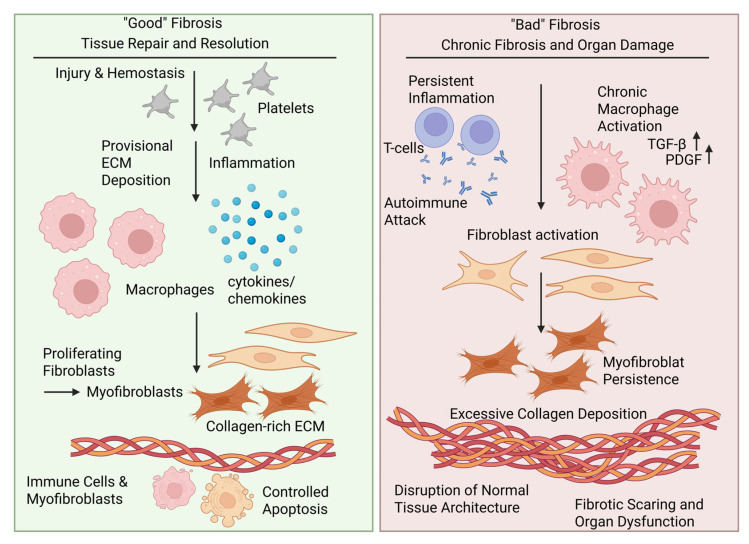
Comparison of “good” fibrosis versus “bad” fibrosis. “Good” fibrosis refers to controlled, reparative ECM remodeling that supports tissue healing, whereas “bad” fibrosis involves excessive, disorganized matrix deposition leading to tissue dysfunction and pathological scarring. Arrows indicate an increase in the concentration of TGF-β and PDGF. (Created using BioRender.com, accessed 28 July 2025).

Fibrosis is often characterized as the degeneration of connective tissue, where excessive accumulation of collagen-rich ECM takes the place of functional tissue [2]. However, the fibrogenic response is a part of the normal wound-healing process, where the deposition and organization of fibrotic scar tissue are essential to restore and maintain organ integrity after injury [2]. Normal repair occurs in a highly regulated sequence of overlapping phases comprising hemostasis, inflammation, proliferation, and remodeling (Figure 1). Wound healing often starts with activated platelets forming a provisional ECM that mechanically stabilizes the damage and immobilizes growth factors, including transforming growth factor beta (TGF-β) and platelet-derived growth factor (PDGF), vascular endothelial growth factor, and fibroblast growth factor [1,2]. Provisional ECM further serves as a migration scaffold for different inflammatory, fibroblastic, and vascular cells to enter the sites of injury. The inflammation that is caused by mast cells and neutrophils protects against invading microorganisms and provides an important source of cytokines. The macrophages that are attracted produce chemokines, like fibroblast growth factors, PDGF, TGF-β1, and vascular endothelial growth factor, which help to recruit more cells within the ECM. Among these attracted cells are fibroblasts, which begin to replace the provisional ECM with fibrillar, collagen-rich ECM of higher mechanical strength [2]. The new ECM mainly contains fibrillar collagen types I and III, basement membrane collagen type IV, and microfibrillar collagen type VI. Fibroblasts begin to transform into contractile myofibroblasts that add further mechanical strength to the scar by remodeling the ECM. Myofibroblasts are strongly positive for α-smooth muscle actin (α-SMA) expression and are characterized by excessive ECM deposition. In time, both the inflammatory cells and myofibroblasts undergo controlled apoptosis to terminate the healing process [3]. Failure of cells to undergo apoptosis is an important factor that can shift the beneficial repair process into harmful organ fibrosis, as the prolonged presence of myofibroblast-activating macrophages and ECM-depositing myofibroblasts leads to abnormal repair and so-called “bad” fibrosis [1,2].

### 1.2. “Bad” Fibrosis—A Pathological Process in Immune-Mediated and Autoimmune Disorders

**Figure 2:**
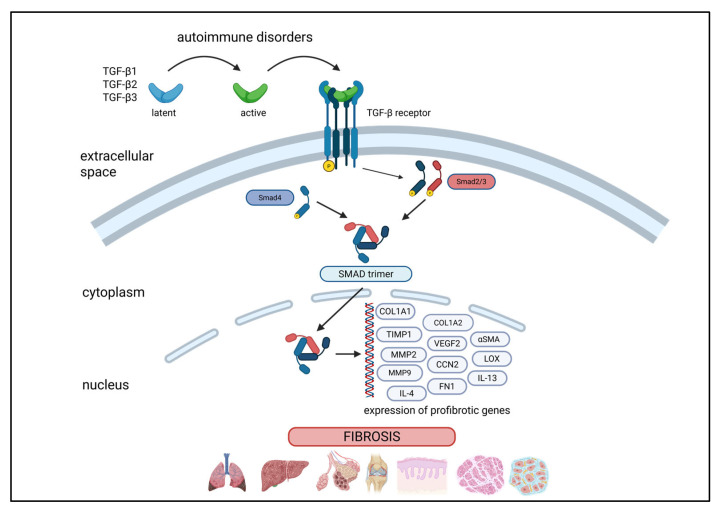
The role of TGF-β signaling in fibrosis. TGF-β is a central mediator of fibrosis that initiates and sustains fibrotic remodeling through both canonical (SMAD-dependent) and non-canonical (SMAD-independent) pathways. Upon ligand binding, TGF-β receptors activate SMAD2/3 proteins, which form complexes with SMAD4 and translocate into the nucleus to regulate the transcription of profibrotic target genes. Simultaneously, TGF-β represses antifibrotic genes and promotes the survival and persistence of myofibroblasts. The resulting gene expression program leads to excessive ECM deposition, tissue stiffening, and scarring. (Created with BioRender.com, accessed 28 July 2025).

“Bad” fibrosis involves the same core mechanisms and pathways as those used in normal tissue repair [1,2]. However, during this process, the fibrotic response shifts from a supportive fibrotic tissue to a microenvironment dominated by an increased number of hyperactivated ECM-producing cells, leading to excessive ECM accumulation, scar formation, and the disruption of normal organ architecture (Figure 1) [1]. TGF-β is a key driver of fibrotic processes, acting through SMAD-dependent and SMAD-independent signaling pathways to regulate gene expression involved in ECM production and fibroblast activation (Figure 2). As the inflammatory cells and myofibroblasts fail to die, the ongoing accumulation and stiffening of scar ECM promote a positive biomechanical feedback loop, maintaining activated cell phenotypes beyond their typical lifespan. In autoimmune diseases, fibrosis is a result of prolonged inflammation, where the immune system targets its tissues. Chronic inflammation triggers fibroblast activation and the deposition of ECM proteins and myofibroblast differentiation, leading to scarring and tissue stiffness. The dysregulation of the immune system in autoimmune diseases creates a vicious cycle, where the inflammation induces fibrosis, and the resulting tissue damage exacerbates immune activation. This loop is seen across a spectrum of autoimmune diseases, although the severity and affected organs may vary.

### 1.3. Fibroblasts as Central Players in Fibrosis

Fibroblasts are spindle-shaped mesenchymal cells ubiquitously present in connective tissues throughout the body. They play a crucial role in maintaining tissue architecture by producing and remodeling the ECM, which provides mechanical support and regulates cellular behavior [1]. Under physiological conditions, fibroblasts contribute to tissue homeostasis and repair, responding dynamically to injury by migrating to the wound site, proliferating, and depositing ECM components such as collagen and fibronectin [4,5]. Fibroblasts are primarily responsible for the synthesis and turnover of ECM proteins, which maintain tissue integrity. They regulate ECM homeostasis through a balance between the secretion of matrix metalloproteinases (MMPs), enzymes that degrade ECM, and tissue inhibitors of metalloproteinases (TIMPs). Beyond structural support, fibroblasts interact closely with immune cells by producing and responding to inflammatory cytokines [1]. In response to persistent tissue damage or chronic inflammation, fibroblasts can differentiate into myofibroblasts [3]. Activated myofibroblasts, which express α-smooth muscle actin, can originate from various stromal progenitor cells, not only fibroblasts, but also from pericytes, endothelial cells (via endothelial-to-mesenchymal transdifferentiation), epithelial cells (via epithelial-to-mesenchymal transdifferentiation), circulating fibrocytes, bone marrow-derived stem cells, and tissue-resident stem cells. Myofibroblasts are the main drivers of ECM production, deposition, and remodeling [4,5]. Their persistent activation, resistance to apoptosis, and altered metabolism lead to excessive accumulation of fibrillar collagens, elastin, fibronectin, and other ECM components, replacing normal tissue architecture with stiff, avascular scar tissue.

Pathogenic fibroblast activation initiates a network of intracellular signaling pathways that sustain and amplify fibrotic processes. Central to this activation is TGF-β, which signals through both canonical SMAD-dependent and non-canonical pathways [4,6]. The non-canonical routes engage signal transducer and activator of transcription 3 (STAT3), non-receptor tyrosine-protein kinases (TYKs), and chromatin-modifying enzymes such as histone acetyltransferase p300, leading to transcriptional reprogramming of fibroblasts [4]. Connective tissue growth factor (CTGF, also known as CCN2) acts as a necessary cofactor for TGF-β–mediated ECM production, sustaining fibrotic responses in both normal and diseased tissue [6]. Other important components of the profibrotic milieu include platelet-derived growth factor (PDGF) and IL-6, which engage Janus kinase (JAK)–STAT pathways. Oxidative stress, generated by tissue hypoxia and reactive oxygen species (ROS) from NADPH oxidase 4 (NOX4) and mitochondria, further enhances these signals, triggering the production of pro-inflammatory cytokines [4]. Fibroblasts also respond to mechanical forces in the fibrotic microenvironment via integrin-mediated mechanotransduction, activating cascades involving focal adhesion kinase (FAK), RHO-associated kinase (ROCK), myocardin-related transcription factors (MRTFA/B), and Yes-associated protein (YAP) [4]. Additional activation cues include thrombin, lysophosphatidic acid (LPA), and endothelin-1 (ET1), which promote fibroblast proliferation, myofibroblast differentiation, extracellular matrix production, and vascular remodeling. Aberrantly reactivated developmental morphogens, including WNT, Hedgehog, Jagged, and Notch, also contribute to sustained activation of fibroblasts [4,6]. WNT proteins activate fibrotic responses via the canonical β-catenin pathway, acting both directly on fibroblasts and indirectly by modulating TGF-β signaling. Hedgehog binds to its receptor Patched 1 (PTC) in the primary cilium, activating GLI1, which drives transcriptional programs that promote fibrosis. Notch signaling is triggered when ligands such as Jagged1, Jagged2, or Delta-like protein 1 (DLK1) bind to the NOTCH1 receptor, leading to activation of target genes through the release of the NOTCH intracellular domain (NICD). Moreover, fibroblasts chemically sense damage-associated molecular patterns (DAMPs) such as tenascin C and fibronectin-EDA in the extracellular matrix, which act as endogenous Toll-like receptor ligands [4].

Fibroblasts are also key mediators of immune–fibrotic crosstalk. Type I interferons (IFNs) act on fibroblasts to increase the production of profibrotic chemokines and to upregulate Toll-like receptors (TLRs), establishing a positive feedback loop that amplifies inflammatory and fibrotic signaling [5]. IFN stimulation further promotes the expression of α-SMA, CTGF, TGF-β2, and ET-1, while downregulating Fli1 and vascular endothelial cadherin, contributing to enhanced vessel permeability and linking fibroblast activation to vascular dysfunction and tissue ischemia.

The convergence and extensive crosstalk between these biochemical and biomechanical pathways drive persistent myofibroblast differentiation, resistance to apoptosis, and ongoing extracellular matrix remodeling, leading to chronic, non-resolving fibrosis. Understanding the complex network of fibroblast activation and persistence is essential, as these processes underpin the pathological fibrosis observed across a range of immune-mediated and autoimmune diseases.

In this review, we explore the mechanisms of fibrosis and its role in several immune-mediated and autoimmune diseases, including systemic sclerosis (SSc), morphea, autoimmune hepatitis (AI), systemic lupus erythematosus (SLE), Sjögren’s syndrome (SS), inflammatory bowel disease (IBD), and rheumatoid arthritis (RA), and discuss emerging antifibrotic therapies.

## 2. Systemic Sclerosis (SSc)

### 2.1. Clinical Presentation and Epidemiology of SSc

SSc is an orphan disease characterized by autoimmunity, fibrosis of the skin and internal organs, and vasculopathy [7]. It is clinically categorized into limited (lcSSc) and diffuse (dcSSc) types, each of which demonstrates different patterns of skin involvement, systemic symptoms, and prognosis [8]. The disease is rare; however, it is often diagnosed too late, and the mortality rate is the highest among all rheumatic diseases, with the overall 10-year survival rate around 60–80%. The annual incidence of SSc is estimated to be between 0.6 and 5.6 per 100,000 adults, and the prevalence between 7.2 and 44.3 per 100,000 adults [8]. The mean age at diagnosis is 33.5 to 59.8 years; pediatric SSc represents no more than 5% of all cases.

SSc is a clinically heterogeneous autoimmune disease characterized by widespread fibrosis, vasculopathy, and immune dysregulation, affecting multiple organ systems. Patients may present with a combination of cutaneous, vascular, internal organ, and constitutional symptoms [8]. Skin abnormalities often appear early in the disease course, beginning with inflammatory edema of the fingers (“puffy fingers”), which progresses to dermal and subcutaneous fibrosis and atrophy, ultimately resulting in sclerodactyly, a pathognomonic feature of SSc. Orofacial fibrosis leads to reduced oral aperture, diminished facial expression (amimia), xerostomia, and sicca syndrome, all of which significantly impair oral and dental health. Rapidly progressing diffuse cutaneous fibrosis is a key predictor of early internal organ involvement [8]. Cutaneous vasculopathy, including Raynaud’s phenomenon (RP), frequently precedes other symptoms by years and is often the initial manifestation. When accompanied by puffy fingers and abnormal nailfold capillaries, it serves as an important diagnostic clue. Digital ulcers, which are usually ischemic, though sometimes triggered by trauma or calcinosis, cause considerable pain and functional impairment. Telangiectasias on the face, palms, and chest reflect underlying systemic microvascular damage. Musculoskeletal involvement includes arthralgia, joint stiffness, erosive arthritis, tendon friction rubs, and fibrosing myopathy with muscle atrophy and weakness [9]. Respiratory complications include interstitial lung disease (ILD) and pulmonary arterial hypertension (PAH); ILD affects approximately 50–65% of patients and is the leading cause of mortality, while PAH, caused by fibrosis of pulmonary vessels, has a 3-year mortality of 21–48% [7]. Cardiac fibrosis can result in myocarditis, heart failure, arrhythmias, pericardial effusions, and rarely, valve sclerosis, all of which significantly increase mortality risk [7,8]. In the kidneys, scleroderma renal crisis (SRC) presents with malignant hypertension, edema, and electrolyte disturbances, occurring in 1–14% of patients and rapidly progressing to end-stage renal disease without prompt treatment, with chronic interstitial and vascular fibrosis contributing to long-term dysfunction [7,8]. Gastrointestinal fibrosis leads to widespread dysmotility, manifesting as reflux, dysphagia, pseudo-obstruction, malabsorption, and fecal incontinence, and is further compounded by salivary gland fibrosis and xerostomia, which impair nutrition [8,9]. Urologic complications, including sexual dysfunction, also reflect the systemic fibrotic burden of the disease.

### 2.2. Pathogenesis of Fibrosis in SSc

The disease is triggered by environmental factors in genetically susceptible individuals through epigenetic modification. Postulated triggers include infection, toxins, immune-mediated cytotoxicity, oxidative stress, anti-endothelial antibodies, and ischemia–reperfusion injury [8]. The pathogenesis of SSc is complex and multifactorial, involving a combination of vascular dysfunction, immune system activation, and progressive fibrosis [10,11]. Vasculopathy is central to the pathogenesis of SSc, including endothelial cell (EC) damage, dysfunction, and defective vascular remodeling [12]. Autoantibodies and immune cells, including anti-endothelial cell antibodies and T cells, are present before clinical symptoms appear, targeting endothelial cells in small blood vessels [11]. However, the precise mechanisms of initial vascular injury remain unclear. Endothelial cells, damaged by autoimmunity and environmental factors, either die or become abnormally activated, triggering inflammation, tissue fibrosis, and impaired vascular repair. The resulting vascular pathology is driven by dysregulated angiogenesis, defective vasculogenesis, and endothelial dysfunction, leading to capillary dilation, loss, and arteriolar stenosis. Activated endothelial cells overexpress adhesion molecules, including GlyCAM-1, ICAM-1, E-selection, and P-selection, promoting the infiltration of Th2/Th17 cells, macrophages, and mast cells, while SSc immune cells upregulate molecules, including L-selectin, integrin α6, and P-selectin, enhancing their attachment to the endothelium. Additional features include the endothelial-to-mesenchymal transition (EndoMT), contributing to fibrosis, and platelet activation with a pro-thrombotic state due to imbalances in vasoconstrictors, vasodilators, and coagulation factors. These processes collectively impair peripheral circulation, perpetuate inflammation, and drive progressive vascular and fibrotic damage in SSc [11].

Immune dysregulation plays a central role in SSc pathogenesis. Immune cell infiltration, primarily T cells, macrophages, mast cells, and B cells, drives chronic inflammation and fibrosis in affected organs (Table 1). T cell subsets are skewed toward Th2 and Th17 dominance, with impaired Treg function; their associated cytokines correlate with disease stage and activity [11]. B cells in SSc are constitutively activated, exhibiting increased expression of activation markers such as CD19, CD21, costimulatory molecules, and B cell activating factor (BAFF) [13]. Moreover, SSc B cells produce numerous autoantibodies, including well-defined biomarkers such as anti-centromere, anti-Scl-70, and anti-RNA polymerase III antibodies [14]. Innate immune cells like mast cells, M2 macrophages, and plasmacytoid dendritic cells (pDCs) also contribute, particularly through the secretion of fibrotic mediators and type I interferons (IFN-α) through the activation of Toll-like receptor (TLR) 7 and 9. Endothelial injury activates pDCs via TLR7 and TLR9, creating a feedforward loop of IFN-α production, endothelial senescence, and immune activation. This persistent immune stimulation fuels vascular injury and fibrosis, positioning immune dysregulation as a central driver in SSc pathogenesis.

Fibroblast activation marks the final step of the disease cascade, leading to excessive ECM production and irreversible organ fibrosis (Table 1). Myofibroblasts, derived from multiple sources including resident fibroblasts, fibrocytes, EndoMT, and adipocyte transdifferentiation, are central to this process [11,14]. TGF-β, especially in early disease, is a key driver of fibroblast activation, inflammation, and ECM production, including fibrillar collagens. In later stages, SSc fibroblasts become autonomously activated through autocrine TGF-β signaling and the increased expression of TGF-β activators like integrins [11]. These fibroblasts are resistant to anti-fibrotic signals from Th1 and Th2 cells, partly due to high progranulin expression. They also shape immune responses by inducing Th2-like Tregs and suppressing IFN-γ through galectin-9, reinforcing a pro-fibrotic microenvironment. Additionally, agonistic autoantibodies against platelet-derived growth factor receptor (PDGFR), angiotensin II type 1 receptor (AT1R), and endothelin A receptor (ETaR) have been implicated in the further activation of fibroblasts and immune cells, correlating with severe SSc complications such as ILD and PAH. Together, these mechanisms form a self-perpetuating fibrotic loop central to SSc pathology.

The shared pathological cascade is further influenced by various organ-specific modifying factors [11]. For example, in the skin, keratinocytes and adipocytes modulate fibroblast activation and influence the development of fibrosis [11]. In the heart, microvascular abnormalities such as capillary loss and arteriolar stenosis lead to tissue hypoxia, which promotes the inflammation and overproduction of extracellular matrix by cardiac fibroblasts, contributing to SSc-related cardiomyopathy [11,15]. In the kidney, fibrosis arises from injuries to glomerular or tubulointerstitial structures and is further exacerbated by early vascular changes such as endothelial damage, thrombosis, and proliferative vasculopathy, which reduce renal perfusion, activate the renin–angiotensin system, and promote perivascular fibrotic remodeling [11,15]. Pulmonary fibrosis pathogenesis involves alveolar epithelial cell injury, activation of pulmonary fibroblasts into matrix-producing myofibroblasts, and a self-perpetuating cycle of epithelial damage and fibroblast activation, primarily driven by TGF-β signaling and interstitial pericytes [15]. The common SSc-specific fibrotic cascade in ILD can be further exacerbated by the microaspiration of gastric contents due to gastroesophageal reflux disease (GERD) [11]. SSc-PAH is primarily caused by arteriolar narrowing due to occlusive vascular fibrosis, while other forms of PH in SSc are linked to cardiac involvement and ILD. These distinct pathological mechanisms often coexist to varying degrees, collectively contributing to elevated pulmonary arterial pressure. The common fibrotic cascade also affects the gastrointestinal tract, leading to smooth muscle atrophy and fibrosis, while vascular changes cause tissue hypoxia and enteric nervous system degeneration. The liver is relatively protected from extensive fibrosis compared to other organs in SSc [11].

Autoantibodies are the most commonly used biomarkers in SSc and are most specifically useful for the diagnosis, classification, and prognosis of SSc [13,16]. Over 90% of SSc patients test positive for anti-nuclear antibodies (ANA). Among these, anti-centromere, anti-Th/To, and anti-topoisomerase I antibodies are considered classical markers, present in approximately 60% of patients, and are associated with distinct clinical subtypes. In addition to ANA, a broad spectrum of other autoantibodies is frequently detected in SSc. These include antibodies targeting endothelial and fibroblast components, angiotensin II type 1 receptor (AT1R), endothelin-1 type A receptor (ETaR), PDGFR, and various ECM proteins. More complex autoantibody systems have also been identified, involving G-protein-coupled receptors, growth factors, and their corresponding receptors. Although about 10% of SSc patients are ANA-negative, several novel autoantibodies have been discovered in both ANA-positive and ANA-negative individuals. These include antibodies against eIF2B, RuvBL1/2 complex, U11/U12 RNP, U3RNP, BICD2, Ku, and PM/Scl [16,17]. Some autoantibodies are related to the increased risk of specific organ fibrosis; for example, the anti-RNAP III antibody is significantly associated with the development of SRC [11], while SSc-ILD has been especially associated with anti-topoisomerase I antibody (anti-Scl-70 antibody) [16]. In addition to autoantibodies, several non-antibody protein biomarkers have been identified in SSc, providing further insight into disease mechanisms and potential prognostic indicators [16].

### 2.3. Current and Emerging Therapies in SSc

Therapy in SSc focuses on symptom management, slowing disease progression, and preserving organ function, as there is currently no cure. Treatment strategies vary depending on disease subtype, limited versus diffuse cutaneous, and the extent of organ involvement [9]. Immunosuppressive agents such as mycophenolate mofetil (MMF), methotrexate, and cyclophosphamide (CYC) are commonly used to reduce inflammation and fibrosis, particularly in skin and lung involvement [13]. The early introduction of immunosuppressants in SSc is crucial, as treatment is often less effective during the later fibrotic stages of the disease. Recent treatment recommendations also include hematopoietic stem cell transplantation (HSCT) for patients with rapidly progressive disease and high risk of organ failure [18]. HSCT represents an intensive immunotherapy that targets the autoreactive adaptive immune system, profoundly resetting immune function, reducing inflammation, halting fibrosis progression, and potentially allowing tissue repair. Clinical studies have shown significant improvements in event-free survival following HSCT. However, due to the considerable risks, such as infections, hematological complications, malignancies, and treatment-related mortality, careful patient selection is essential [19]. Concerns also remain regarding the long-term durability of its effects.

Antifibrotic agents approved for pulmonary fibrosis, such as nintedanib and pirfenidone, have been evaluated in SSc-ILD trials (Table 2) [19]. Nintedanib, a tyrosine kinase inhibitor, is approved by both the FDA and EMA for SSc-ILD, based on the SENSCIS trial, which demonstrated its ability to significantly slow lung function decline [20]. Although effective across a range of patient subgroups, it does not improve skin symptoms and is commonly associated with gastrointestinal side effects, particularly diarrhea [13]. Pirfenidone, while not approved for SSc-ILD, has shown a good safety profile when used alongside immunosuppressants and some early signs of efficacy in clinical trials. However, evidence is still limited due to small sample sizes and increased side effects [19].

In addition to potential disease-modifying therapies, it is essential to address exacerbating factors such as GERD and intercurrent infections. For patients with end-stage lung fibrosis, lung transplantation remains the only option that can significantly improve long-term outcomes [19]. Vasodilators, including calcium channel blockers, endothelin receptor antagonists, and phosphodiesterase-5 inhibitors, are used to manage vascular complications such as RP and PAH [9,13]. Recently, biologic therapies have emerged as promising new treatments. Rituximab (RTX), a monoclonal antibody targeting CD20, has shown potential in treating both cutaneous and pulmonary manifestations of SSc by depleting B cells. It appears to have antifibrotic effects, improve inflammation and lung function, and carries a favorable safety profile [13]. Tocilizumab (TCZ), an anti-IL-6 monoclonal antibody, has been evaluated due to IL-6’s central role in SSc pathogenesis. Although TCZ did not significantly reduce skin thickening in clinical trials, it consistently slowed lung function decline in SSc-ILD patients, suggesting it may be beneficial for those at high risk of progression [13].

CAR-T cell therapy is an emerging and highly promising approach. A study of CD19-targeted CAR T-cell therapy in six patients with refractory SSs showed profound improvements in disease activity, with halted fibrosis progression and discontinuation of all prior immunosuppressive and antifibrotic treatments [21]. However, a longer follow-up is needed to determine whether disease progression is sustainably halted.

JAK-STAT inhibitors, such as tofacitinib, also represent an evolving therapeutic avenue (Table 2). A systematic review of 59 patients showed that JAK inhibitors are effective and generally safe for treating skin fibrosis in SSc, both as a first-line option and in refractory cases [22]. Preliminary evidence also suggests potential benefits for SSc-ILD. Other targeted therapies that are being investigated also include Belimumab, a BAFF inhibitor; Abatacept, a CTLA-4–Ig fusion protein that inhibits CD80/CD86–CD28 co-stimulation; Fresolimumab, an anti-TGF-β antibody; and Romilkimab, an anti-IL4 and anti-IL13 antibody [23]. Despite these advances, current treatments largely focus on symptom control and slowing progression, rather than reversing disease or improving survival. This underscores the critical need for continued development of novel, targeted therapies in systemic sclerosis.

## 3. Morphea

### 3.1. Clinical Presentation and Epidemiology of Morphea

Morphea, a localized scleroderma, is distinct from SSc autoimmune connective tissue disorders that, despite sharing certain clinical and pathological features, differ significantly in their pathogenesis, systemic involvement, and disease course. Both conditions are characterized by inflammation, vascular abnormalities, and skin fibrosis driven by dysregulated immune responses and excessive collagen production. However, morphea is typically limited to the skin and underlying tissues, lacking internal organ involvement, and differs in immunologic profiles, clinical management, and prognosis. Morphea is generally characterized by the inflammatory patches and bands of thickened skin on the head, neck, trunk, and extremities of the body, occurring as a result of the abnormal deposition of collagen caused by both inflammatory and fibrotic components [24]. It occurs primarily in children 2–14 years old and exhibits a female predominance [24,25]. The overall incident rate ranges from 4 to 27 new cases per million people, and juvenile localized scleroderma has an occurrence of 3.4 to 9 cases per million children per year, with the peak incidence observed at 10 years old.

### 3.2. Pathogenesis of Fibrosis in Morphea

Morphea can manifest in varied clinical forms, categorized into five main subtypes: limited, generalized, linear, deep, and mixed [24]. It presents as thickened patches or bands of skin affecting the head, neck, trunk, and limbs, resulting from the activation of inflammatory and profibrotic pathways that lead to excessive collagen deposition. Despite extensive research, the precise pathogenesis remains unclear. Contributing factors likely include genetic predisposition, environmental triggers, immune dysregulation with abnormal cytokine release, and vascular abnormalities. Morphea generally progresses through three overlapping stages: an initial inflammatory phase, a fibrotic or sclerotic phase, and a late atrophic phase. Early disease is marked by immune cell infiltration, primarily activated T cells (particularly CD4+), macrophages, plasma cells, and eosinophils, around skin and blood vessels (Table 1). The immune response is dominated by Th1 and Th17 cytokines early on, with a later shift toward Th2 cytokines such as IL-4, IL-6, and TGF-β, which promote fibrosis [24]. Vascular endothelial changes and the upregulation of adhesion molecules like E-selectin and VCAM-1 facilitate leukocyte recruitment and inflammation. TGF-β and IL-4 play central roles in fibroblast activation and collagen overproduction, while vascular and dendritic cell changes also contribute to sclerosis. The final atrophic stage, though poorly understood, may follow years after sclerosis onset and includes skin thinning, persistent collagen deposition, pigmentary changes, and low-grade inflammation. At this point, the inflammatory process subsides, and the disease may become inactive, with tissue remodeling likely influenced by altered matrix metalloproteinase activity regulated by T cells [24].

### 3.3. Current and Emerging Therapies in Morphea

The current treatment options for morphea are limited and often suboptimal. Both topical and systemic therapies are employed, with topical agents including corticosteroids, tacrolimus, vitamin D analogs, and imiquimod [24]. Systemically, methotrexate, either alone or in combination with systemic corticosteroids, is regarded as the first-line treatment, especially in pediatric cases. For patients unresponsive or intolerant to methotrexate, or in cases of severe or recurrent disease, MMF is considered a second-line option. Additional therapeutic agents include cyclosporine, hydroxychloroquine, azathioprine, retinoids, intravenous immunoglobulins, and biologics like rituximab and infliximab, as well as phototherapy-based treatments (Table 2) [24]. Despite the availability of these interventions, managing morphea remains challenging due to the nonspecificity of treatments, risk of significant long-term side effects, and variable patient response, including frequent relapses and refractory cases, particularly in children, who often exhibit more aggressive disease and higher relapse rates even after prolonged remission. Therefore, there is a critical need for new therapies that effectively target early inflammation and prevent fibrosis and tissue atrophy. Recent research has identified potential new targets for therapy, including JAK-STAT inhibitors [26], epigenetic modulators such as BET and HDAC inhibitors [27,28], and biologic agents targeting key fibrotic and inflammatory mediators like TGF-β, CTGF, IL-6, and IL-4 [25,29]. Elevated IL-6 levels in morphea patients have prompted trials of tocilizumab, an anti-IL-6 monoclonal antibody, with intravenous administration leading to improvement in severe pediatric cases, especially in combination with methotrexate [29]. Tofacitinib and baricitinib, targeting the JAK/STAT pathway, have also shown clinical benefits in morphea cases, while abatacept (a CTLA-4-Ig fusion protein) has reduced disease activity and corticosteroid dependence in children and adults. Imatinib, targeting PDGF and c-Abl, has demonstrated antifibrotic effects in generalized morphea [29]. However, most studies remain small or retrospective, highlighting the need for prospective studies and biomarkers to guide therapy.

## 4. Autoimmune Hepatitis (AIH)

### 4.1. Clinical Presentation and Epidemiology of AIH

AIH is a rare and progressive chronic immune-inflammatory liver disease [30]. It is characterized by immune-mediated hepatocyte injury resulting in the destruction of liver cells, causing inflammation, liver failure, and fibrosis [31]. Epidemiological data show that AIH affects children and adults of all ages and ethnicities [32], although the onset of the disease increases with age, with peak incidence in the range of 50 years or older [33]. The global pooled incidence of AIH is estimated to be 1.28 cases per 100,000 inhabitants/year, and the prevalence is 15.65 cases per 100,000 inhabitants, yet with a growing trend worldwide [34].

The clinical presentation of AIH is not characteristic, ranging from totally asymptomatic and mild forms with nonspecific symptoms, like malaise, lethargy, nausea, anorexia, pruritus, and jaundice, to severe hepatitis [30]. It is usually characterized by interface hepatitis on liver histology, hypergammaglobulinemia, elevated aminotransferases, and circulating autoantibodies [30]. There are three main subtypes of AIH based on autoantibody profiles. AIH type 1 is the most common (about 80%) and is typically associated with ANA, smooth muscle antibodies (SMA), and perinuclear ANCA (p-ANCA). AIH type 2, which is rare (3–4%) and usually affects children, is defined by the presence of anti-LKM1, anti-LKM3, and/or anti-LC1 antibodies. AIH type 3 accounts for 10–20% of cases and is marked by antibodies against soluble liver antigen (SLA) or liver-pancreas antigen (LP) [30].

### 4.2. Pathogenesis of Fibrosis in AIH

Although the exact etiology of AIH remains unclear, it is believed that a combination of genetic susceptibility and environmental triggers underlies disease development [32,35]. Genetic predisposition is largely associated with polymorphisms in the human leukocyte antigen (HLA) region, which encodes the major histocompatibility complex (MHC). Environmental triggers may include certain drugs or viral infections, which contribute to the loss of immune tolerance.

In AIH, this immune dysregulation leads to chronic liver inflammation. A key event is the activation of antigen-presenting cells (APCs) in the liver, such as Kupffer cells, liver sinusoidal endothelial cells (LSECs), and dendritic cells, which present hepatic autoantigens via MHC molecules to CD4+ and CD8+ T cells (Table 1) [32,35]. CD4+ T cells differentiate into effector subsets based on cytokine signals: Th1 cells (via IL-12) produce IL-2 and IFN-γ, enhancing cytotoxic T cell and macrophage activation; Th2 cells (via IL-4) aid B cells in producing autoantibodies; and Th17 cells (via TGF-β, IL-6, and IL-1β) release IL-17 and IL-22, amplifying inflammation and hepatocellular injury. Regulatory T cells (Tregs), which normally suppress inappropriate immune responses, are functionally impaired in AIH, further contributing to immune overactivity [32]. Additionally, increased intrahepatic activity of NK cells, memory CD8+ T cells, and CXCR3+ Th1/Th17 cells, along with elevated levels of their chemokine ligand CXCL10, correlates with disease severity and progression toward fibrosis. Molecular mimicry, in which viral antigens resemble hepatic proteins, can confuse the immune system and trigger or exacerbate AIH [32]. Furthermore, recent studies suggest that gut microbiota imbalance (dysbiosis) may modulate immune responses, promoting hepatic inflammation.

Chronic liver inflammation leads to progressive tissue damage and fibrosis. Liver fibrosis is characterized by excessive ECM deposition, primarily collagen (Table 1) [36]. Hepatic stellate cells (HSCs), normally quiescent cells in the space of Disse storing vitamin A, play a central role. Upon liver injury, dying hepatocytes release reactive oxygen species (ROS), damage-associated molecular patterns (DAMPs), and lipid peroxidation products [36]. These activate Kupffer cells, LSECs, and infiltrating immune cells, which in turn release profibrotic cytokines such as TGF-β1, PDGF, tumor necrosis factor-α (TNF-α), and interleukins (e.g., IL-1β, IL-6) [36]. TGF-β1 is a key mediator of fibrogenesis. It activates SMAD2/3 signaling in HSCs, promoting the transcription of collagen type I and III, fibronectin, and other ECM components. PDGF promotes HSC proliferation and migration [36]. TLR4 enhances HSC sensitivity to TGF-β signaling. Meanwhile, matrix metalloproteinases (MMPs), responsible for ECM degradation, are inhibited by tissue inhibitors of metalloproteinases (TIMPs), particularly TIMP-1, tipping the balance toward matrix accumulation. Gut-derived pathogen-associated molecular patterns (PAMPs), especially lipopolysaccharides (LPS), enter the liver via the portal circulation due to increased intestinal permeability. These further activate TLR4 and TLR9 on Kupffer cells and HSCs, driving inflammation and fibrosis. Inflammasomes like NLRP3 also contribute by activating caspase-1, leading to IL-1β and IL-18 maturation. Simultaneously, Wnt/β-catenin signaling supports HSC activation and fibrotic remodeling.

As fibrosis progresses, ECM deposition increases 6–8-fold [37]. Type IV collagen is replaced by fibrogenic types I and III. Activated HSCs also express α-SMA and secrete fibronectin and hyaluronic acid. Accumulated ECM in the space of Disse leads to sinusoidal capillarization and impaired hepatocyte–blood exchange [38]. Bridging fibrosis forms between portal tracts and central veins, eventually producing regenerative nodules surrounded by scar tissue, a hallmark of cirrhosis [39]. These architectural distortions compromise hepatic function and contribute to portal hypertension and liver failure. The differential diagnosis of AIH is broad and varied, requiring careful integration of clinical presentation, laboratory results (i.e., elevated serum transaminases and increased serum IgG), and liver histology to confirm the diagnosis [40]. Serological testing for autoantibodies is essential, but approximately 10% of cases may be seronegative, potentially leading to underdiagnosis. Therefore, liver biopsy remains necessary to confirm the diagnosis and assess characteristic histological features. However, while liver biopsy remains the “gold standard” for evaluating the extent of hepatic fibrosis in AIH, its invasive nature and potential for serious complications limit its repeated use. As an alternative, noninvasive assessment methods, such as serum biomarkers and imaging techniques, have been proposed. The aspartate aminotransferase-to-platelet to-platelet ratio index (APRI), fibrosis-4 index (FIB-4), and FibroTest, which combines five serum markers (α2-macroglobulin, haptoglobin, apolipoprotein A1, γ-glutamyl transpeptidase, and total bilirubin), are noninvasive markers of liver fibrosis, but they were demonstrated with only moderate sensitivity and specificity in AIH [30,41]. The most promising seems to be the serum ferritin–zinc ratio, which has recently been shown to predict liver fibrosis progression in AIH [42]. There are also studies showing the potential of circulating microRNAs, including miR-122, miR-21, and miR-155 [43,44], circulating proteins, like CA1, CA3, GAS6, FCGR2A, 4E-BP1, and CCL19 [31], or growth differentiation factor 15 (GDF15) [45]; however, they require more studies. Imaging-based noninvasive tools for AIH diagnosis include transient elastography (TE), MRI-based techniques, like magnetic resonance elastography (MRE) and magnetic resonance spectroscopy (MRS), and ultrasound-based elastography [30].

### 4.3. Current and Emerging Therapies in AIH

The first-line therapy for AIH typically involves a combination of corticosteroids (prednisolone) and the immunosuppressant azathioprine (AZA) (Table 2) [30]. Steroid-related adverse effects such as weight gain, osteoporosis, diabetes, and hypertension can be mitigated by switching to budesonide, especially in non-cirrhotic patients, as it offers fewer side effects and higher remission rates. AZA intolerance, often associated with cirrhosis, may manifest as arthralgia, fever, or pancreatitis, and in such cases, MMF is a recommended alternative. MMF is effective in AZA-intolerant and even treatment-naïve patients, though its high cost and teratogenicity limit its use. For incomplete or non-responders, second- and third-line options include calcineurin inhibitors (Cyclosporine A, Tacrolimus), mTOR inhibitors (Everolimus), and monoclonal antibodies (Rituximab, Infliximab) [30,32], yet the latter are reserved only for highly refractory cases.

AIH, if left untreated, can progress to fibrosis, cirrhosis, and liver failure, highlighting the need for effective therapeutic strategies. With standard treatments, many patients experience relapses, treatment intolerance, or insufficient response. In recent years, adult stem cells (ASCs) have emerged as promising candidates for regenerative and immunomodulatory therapies in AIH [46]. Hematopoietic stem cells, originally used in hematological malignancies, have demonstrated beneficial effects in autoimmune diseases like multiple sclerosis and are being explored for their capacity to modulate immune responses in AIH. Mesenchymal stem cells (MSCs), derived from sources such as bone marrow, adipose tissue, umbilical cord, and dental pulp, exhibit strong immunosuppressive effects through the secretion of soluble factors and cell–cell interactions that modulate T cells, B cells, dendritic cells, and macrophages. Preclinical studies have shown that MSCs can attenuate liver inflammation, reduce hepatocellular damage, and inhibit hepatic stellate cell activation, potentially reversing fibrosis. Furthermore, ASC-derived exosomes, nano-sized vesicles carrying bioactive molecules, have shown similar immunoregulatory and anti-fibrotic effects, offering a cell-free therapeutic approach [46,47]. Ongoing research continues to assess the safety, efficacy, and mechanisms of ASC-based therapies in AIH, which may complement or eventually serve as alternatives to conventional treatments, particularly in refractory cases [46].

Several targeted therapies for liver fibrosis are also under investigation, aiming to reduce hepatocyte apoptosis (e.g., Emricasas—pan-caspase inhibitor, Selonsertib—ASK1 inhibitor), oxidative stress (e.g., resveratrol, NOX1/4 inhibitor), HSC activation (e.g., obeticholic acid—FXR agonist), and immune-driven inflammation (e.g., Cenicriviroc—CCR2/CCR5 inhibitor, Belapectin—galectin-3 inhibitor) [36]; however, they have not been tested strictly in AIH. The targeted therapies under investigation in AIH include, among others, Zetomipzomib, a small-molecule, selective immunoproteasome inhibitor that modulates multiple immune functions (Table 2). These include the reduced production of proinflammatory cytokines, decreased activity of inflammatory T helper 1 and 17 (Th1/Th17) cell subsets, increased numbers of regulatory T cells, and reduced plasma cell and autoantibody production [40]. Another investigational drug is Ianalumab, a monoclonal antibody targeting the BAFF receptor expressed on B cells, which has been shown to be overexpressed in AIH [40]. JKB-122, a TLR4 antagonist, is currently under investigation, as TLR2/4 ligands are known to induce the release of large amounts of proinflammatory cytokines, leading to rapid apoptosis and necrosis of hepatocytes. Studies in a mouse model have demonstrated a significant reduction in proinflammatory cytokines in both serum and liver following treatment [40]. JAK inhibitors have, so far, not been widely studied in AIH; only a few case reports have been published, suggesting their potential usefulness, similar to the possible application of anti-IL-1 and anti-IL-6 therapies [40,48]. Therapies with promising outcomes also include adoptive Treg transfer and low-dose IL-2 administration to increase Treg numbers, with both aimed at restoring functionally impaired Tregs in AIH [35].

## 5. Systemic Lupus Erythematosus (SLE)

### 5.1. Clinical Presentation and Epidemiology of SLE

SLE is a multifactorial, chronic autoimmune disorder characterized by pervasive immune dysregulation and multisystem inflammation [49]. Historical accounts of SLE date back to approximately 400 B.C [50].

The disease is defined by a breakdown in immunological self-tolerance, leading to the immune system erroneously targeting endogenous tissues [51]. This results in sustained inflammatory responses and organ damage involving the skin, kidneys, lungs, central nervous system, joints, and vasculature [52].

The etiopathogenesis of SLE is primarily driven by an aberrant immune response in genetically predisposed individuals, precipitated by environmental triggers such as microbial infections, hormonal fluctuations, and exposure to ultraviolet (UV) radiation. A key pathogenic mechanism involves the defective clearance of apoptotic cells, leading to the persistence of nuclear self-antigens that potentiate chronic immune activation [53].

Abnormalities in the innate immune system, particularly excessive type I IFN signaling, are central to both the initiation and amplification of inflammatory cascades. These innate immune responses subsequently engage and dysregulate components of the adaptive immune system. Notably, B lymphocyte hyperactivity in SLE leads to the production of pathogenic autoantibodies, including ANAs and anti-Smith (anti-Sm) antibodies [54]. These autoantibodies form immune complexes that deposit in tissues, initiating complement activation and resulting in end-organ damage. T lymphocyte dysfunction, characterized by impaired regulatory T cell activity and aberrant cytokine production—particularly elevated IFN-α—further exacerbates immunopathology [55].

Genomic studies, particularly genome-wide association studies (GWAS), have identified more than 100 susceptibility loci associated with SLE. These loci predominantly affect genes involved in immune regulation, antigen processing and presentation, and type I IFN signaling pathways [56]. Emerging evidence has illuminated additional molecular pathways implicated in specific clinical manifestations of SLE. For example, the Hippo signaling pathway is hyperactivated in keratinocytes upon UVB exposure, enhancing apoptotic processes and contributing to photosensitive skin lesions. Moreover, the increased expression of apoptosis-related pathways, such as Fas/Fas ligand (Fas/FasL) interactions, has been observed in T cells derived from individuals with SLE, further contributing to immunopathogenesis [57].

SLE is characterized by marked clinical heterogeneity, presenting a considerable challenge for both diagnosis and therapeutic management. This variability arises from the multifaceted involvement of multiple organ systems and underlying genetic diversity among affected individuals.

Historically, therapeutic interventions have predominantly employed broad-spectrum immunosuppressive agents, including corticosteroids, non-steroidal anti-inflammatory drugs (NSAIDs), cytotoxic agents, and antimalarial medications. Although these treatments are effective in attenuating disease activity, they are not curative and are frequently associated with significant adverse effects [58].

The heterogeneity of clinical manifestations and pathophysiological mechanisms complicates the standardization of clinical trial designs and highlights the necessity for individualized treatment strategies. Current research efforts are focused on the development of targeted therapeutic modalities with enhanced safety and efficacy profiles. In particular, the long-term evaluation of combination therapies is ongoing to achieve more durable disease control and minimize treatment-related toxicity [59].

SLE is estimated to affect approximately 3.4 million individuals worldwide, with an annual incidence of roughly 400,000 new cases [60]. The disease exhibits a pronounced female predominance, particularly during the reproductive years, with incidence rates increasing markedly between puberty and menopause [61]. While the female-to-male ratio is approximately 3:1 in pediatric populations, this disparity widens significantly in adults, ranging from 9:1 to 15:1 during peak reproductive age [62].

Ethnicity plays a pivotal role in modulating both the susceptibility to and clinical phenotype of SLE. Individuals of African ancestry—especially those residing in North America or Europe—are disproportionately affected, often presenting with earlier disease onset and higher prevalence compared to individuals of Northern European descent [63]. Similarly, populations of Chinese and Hispanic origin demonstrate elevated disease risk and an increased incidence of severe complications such as lupus nephritis (LN), a major renal manifestation of SLE that is closely associated with heightened morbidity and mortality.

A comprehensive global epidemiological study conducted in 2023 identified Poland, the United States, Barbados, and China as countries with notably elevated SLE incidence rates [60]. Regional and ethnic variations in disease prevalence are multifactorial, influenced by disparities in healthcare access, socioeconomic conditions, environmental exposures (e.g., infections), prevalence of comorbidities such as cerebrovascular disease, and adherence to therapeutic regimens.

SLE is a clinically and immunologically heterogeneous autoimmune disorder, characterized by considerable interindividual variability in disease onset, activity, and organ system involvement. Accurate diagnosis and clinical evaluation typically depend on standardized classification systems such as the American College of Rheumatology (ACR) criteria, alongside validated disease activity measures, including the Systemic Lupus Erythematosus Disease Activity Index (SLEDAI) [64].

### 5.2. Pathogenesis of Fibrosis in SLE

Fibrosis in SLE results from persistent immune activation, chronic inflammation, and dysregulated tissue repair mechanisms. The pathological accumulation of ECM components leads to tissue scarring and organ dysfunction. Fibrotic remodeling may develop in multiple organs affected by systemic lupus erythematosus, including the kidneys, lungs, skin, and heart. These fibrotic alterations contribute significantly to chronic morbidity and may reduce responsiveness to therapeutic interventions [65].

Renal fibrosis is a multifactorial and maladaptive pathological response triggered by diverse injurious stimuli, affecting either the glomerular or tubular structures of the kidney. This fibrotic process constitutes a common terminal pathway that underlies the progression of chronic kidney disease (CKD) and ultimately leads to end-stage renal disease (ESRD) [66]. Fibrotic changes have been extensively observed in LN, a severe renal manifestation of SLE. LN occurs in approximately 40% of adults and up to 80% of pediatric patients diagnosed with SLE [65]. Despite therapeutic advances, LN continues to be a major contributor to morbidity and mortality, with around 10% of affected individuals advancing to ESRD and requiring renal replacement therapies, such as dialysis or kidney transplantation [67].

Pulmonary fibrosis (PF) is a rare but serious complication of systemic lupus erythematosus. About 15% of SLE patients develop ILD, particularly nonspecific interstitial pneumonia (NSIP) [68]. Pulmonary fibrosis is characterized by chronic, progressive scarring of the lung parenchyma, leading to impaired gas exchange and reduced respiratory function [69]. Clinically, pulmonary fibrosis presents with exertional dyspnea, nonproductive cough, and bibasilar crackles. High-resolution computed tomography (HRCT) typically reveals a reticular pattern with honeycombing, especially in subpleural and basal regions [69].

Multiple cellular and molecular mechanisms contribute to the development of fibrosis in SLE, with notable roles played by myofibroblasts, the epithelial-to-mesenchymal transition (EMT), macrophages, TGF-β, and neutrophil extracellular trap (NET) formation pathways (Table 1).

Myofibroblasts are widely recognized as central mediators of fibrotic processes across various organs, including the kidneys and lungs [15]. These cells synthesize collagen and other ECM components to facilitate tissue repair following injury. Under normal conditions, myofibroblasts undergo apoptosis once tissue integrity is restored. However, in the context of persistent injury or chronic inflammation, they remain activated, leading to sustained ECM production and progressive fibrosis (Table 1). Additionally, myofibroblasts can promote oxidative stress through reactive oxygen species (ROS) generation and modulate cellular proliferation via PDGFs [70]. The differentiation of myofibroblasts often represents the culmination of EMT, a dynamic process in which epithelial cells gradually acquire mesenchymal characteristics, including enhanced motility and altered secretory profiles [71].

Cells undergoing the EMT contribute to the fibrotic microenvironment by releasing pro-inflammatory and pro-fibrotic cytokines, such as TGF-β, IL-6, and TNF-α, which in turn stimulate the activation and differentiation of interstitial fibroblasts into myofibroblasts (Table 1) [71]. One well-characterized regulatory axis implicated in EMT is the Wnt/β-catenin signaling pathway, which has been shown to promote the mesenchymal transition and fibrogenic activity [72].

Macrophages represent another cellular component implicated in the pathogenesis of renal fibrosis, where they constitute a dominant component of the inflammatory infiltrate. Distinct macrophage phenotypes exert divergent roles in the initiation and progression of LN. Classically activated macrophages (M1) are characterized by their pro-inflammatory profile, including the expression of inducible nitric oxide synthase (iNOS) and the secretion of cytokines such as IL-1β, TNF-α, and IL-6 [73]. These cells contribute to the amplification of inflammation and tissue damage. In contrast, alternatively activated macrophages (M2) are associated with anti-inflammatory activities. They exhibit elevated levels of arginase-1, mannose receptor (MR), and IL-10, which are indicative of their role in promoting the resolution of inflammation and tissue regeneration [73]. The presence of mixed phenotype macrophages within the renal parenchyma suggests an imbalance in macrophage polarization and is associated with poor clinical outcomes in LN [74]. The polarization states of macrophages are tightly regulated by various metabolic pathways, including glycolysis, the pentose phosphate pathway, amino sugar and nucleotide sugar metabolism, fatty acid oxidation, sphingolipid metabolism, the tricarboxylic acid (TCA) cycle, arginine metabolism, and tryptophan metabolism, all of which influence macrophage function and fate [74].

The TGF-β/Smad signaling pathway plays a critical role in the pathogenesis of fibrosis across multiple organs, including the kidneys, lungs, and heart (Figure 2) [75]. TGF-β1 activates the pathway by inducing the phosphorylation of Smad2 and Smad3, two receptor-associated Smads (R-Smads).

Subsequently, they activate transcription factors that promote the production of ECM components, such as collagen and glycoproteins, thereby increasing ECM deposition. Additionally, they inhibit the activity of metalloproteinases, reducing ECM degradation [76]. Elevated levels of TGF-β1 have been reported in various experimental models and clinical cases of CKD, implicating this cytokine as a central mediator of renal fibrosis [77]. One of the downstream consequences of TGF-β/Smad activation is the promotion of EMT, wherein renal epithelial cells acquire a mesenchymal phenotype and contribute to the expanding population of myofibroblasts. Additionally, persistent TGF-β signaling has been associated with structural damage in the kidney, including tubular atrophy, podocyte loss, and rarefaction of the peritubular capillary network, all of which exacerbate tissue hypoxia and drive hypoxia-induced fibrotic responses. The upregulation of TGF-β expression has been consistently observed in both glomerular and tubular compartments in fibrotic kidneys under various pathological conditions [76].

Neutrophils are rapidly recruited to sites of tissue injury, where they can undergo a specialized form of programmed cell death known as NETosis, leading to the release of NETs. While NETs serve an essential antimicrobial function by trapping and neutralizing pathogens, their dysregulated release has been implicated in exacerbating tissue injury and promoting inflammatory responses [78]. In addition to their immunological role, NETs have been shown to influence fibroblast activation and differentiation [79]. Emerging evidence has demonstrated elevated NET levels in the pulmonary tissue of patients with SLE [80]. Comprehensive transcriptomic profiling suggests that NET formation is associated with the induction of EMT [80]. In vitro experiments demonstrated that NET stimulation significantly increases the expression of mesenchymal markers such asα-SMA, Twist, and Snail, while concurrently reducing the expression of the epithelial marker E-cadherin [80]. These findings suggest that the NETs-EMT axis may play a contributory role in the pathogenesis and progression of pulmonary fibrosis.

### 5.3. Current and Emerging Therapies in SLE

Conventional therapeutic approaches in SLE primarily aim to suppress the immune response and control systemic inflammation. However, these strategies often fall short in preventing or reversing the progression of fibrosis once it is established. Current clinical management of fibrotic complications in SLE involves the use of broad immunosuppressive agents, such as corticosteroids, MMF, and CYC (Table 2). In addition, newer biologic agents like belimumab and anifrolumab, which target specific immune signaling pathways, have shown promise in modulating disease activity. Recently, attention has shifted toward antifibrotic therapies originally developed for other fibrotic disorders. Nintedanib, a multi-tyrosine kinase inhibitor approved for idiopathic pulmonary fibrosis (IPF), demonstrated efficacy in reducing lung function decline and acute exacerbations in patients with IPF, as shown in the INPULSIS trials (Table 2) [81]. This compound inhibits signaling through PDGF receptors, fibroblast proliferation, and the differentiation of fibroblasts into myofibroblasts. Preclinical models have shown that nintedanib attenuates fibrosis through the suppression of pro-fibrotic mediators such as IL-1β, keratinocyte chemoattractant (KC), TIMP-1, and collagen deposition in the lungs [82]. Histological assessment in murine models further supports its role in reducing inflammatory infiltration, granulomatous lesions, and fibrotic tissue remodeling.

Efforts to interfere with the TGF-β pathway—a central driver of fibrosis—have included the development of neutralizing antibodies [83], receptor-specific small molecule inhibitors [84], interventions targeting latent TGF-β complexes [85], and antisense oligonucleotides targeting TGF-β1 mRNA (Table 2) [86]. Nevertheless, these approaches have been met with limited success in clinical translation due to adverse effects and the complexity of TGF-β signaling regulation [87].

Emerging evidence suggests that mesenchymal stem cell (MSC) therapy may offer a novel strategy for addressing fibrosis in autoimmune diseases (Table 2). In particular, pretreatment of human umbilical cord-derived MSCs (hUC-MSCs) with dimethyloxallyl glycine (DMOG) has been shown to enhance their therapeutic potential in a murine lupus model (MRL/lpr mice) [88]. DMOG-treated MSCs exhibited improved homing and tissue penetration capabilities, alongside a reduction in lymphoid organ hyperplasia. Furthermore, these cells contributed to the preservation of renal architecture, suppressed inflammatory infiltration, and mitigated fibrotic changes. Molecular analysis revealed decreased expression of fibrotic markers such as fibronectin, collagen type I alpha 1 (Col1a1), collagen type III alpha 1 (Col3a1), and proinflammatory cytokines, including TNF-α, IFN-γ, and IL-6.

Despite these advances, there remains a lack of approved therapies specifically targeting fibrotic manifestations in SLE. This underscores an urgent need for the development of targeted antifibrotic agents that are both effective and safe in the context of autoimmune pathology.

## 6. Sjögren’s Syndrome (SS)

### 6.1. Clinical Presentation and Epidemiology of SS

SS is a chronic systemic autoimmune condition distinguished by immune-mediated damage to the salivary and lacrimal glands, resulting in symptoms such as dry mouth (xerostomia) and dry eyes (xerophthalmia). This syndrome is classified as “primary” when it occurs independently and “secondary” when it is associated with other autoimmune connective tissue disorders, including systemic lupus erythematosus, rheumatoid arthritis, or systemic sclerosis.

From a serological perspective, the presence of ANA, rheumatoid factor (RF), and specific autoantibodies such as Ro/SSA and La/SSB is commonly observed in individuals with SS [89].

The pathogenesis of SS is multifaceted, involving both genetic susceptibility and environmental influences. Various infectious agents, particularly viruses, have been proposed as potential triggers for the development of SS. Notably, Epstein–Barr virus (EBV) has been identified in biopsies of the lacrimal glands, as well as in saliva and specimens from salivary glands [90]. EBV is known to activate the innate immune response, which enhances the expression of IFN through interactions with endosomal TLRs [91].

Genetic factors are believed to significantly contribute to the pathogenesis of SS. Genomic studies have demonstrated associations between specific HLA alleles, such as DRB103:01, DQA105:01, and DQB1*02:01, and an increased susceptibility to SS [92]. Furthermore, variants in the IRF5 and STAT4 genes have been linked to the condition, suggesting their involvement in regulating the type II IFN signaling pathway [93].

Epithelial cells are recognized as critical contributors to the pathogenesis of SS [94] as they not only serve as targets for the autoimmune attack but also play a pivotal role in initiating immune activation [94]. Epithelial cells exhibit diverse regulatory roles, which encompass the expression of ribonucleoprotein complexes, facilitating interactions with T cells via surface costimulatory proteins such as CD86. They also produce cytokines, including IL-21 and BAFF, which play a critical role in the differentiation of Tfh cells. These Tfh cells, in turn, regulate B-cell function. Additionally, they secrete chemokines such as CXCL12, which facilitate the recruitment of leukocytes to the target site [95,96].

Additionally, local inflammatory responses and the presence of cytokines, including IFN-γ and TNF-α, may disrupt the integrity of tight junctions within epithelial cells, resulting in modifications to cell polarity and structural organization [97]. These alterations can lead to a decrease in both the quality and quantity of saliva, thereby intensifying the inflammatory processes observed in the salivary glands of individuals with SS.

SS predominantly affects women, with a female-to-male ratio of approximately 9:1, particularly among individuals of middle age [98]. The diagnosis of SS typically occurs during the fifth decade of life, with a mean age ranging from 51.6 (±13.8) to 62 (±13) years [99]. The incidence of SS is estimated at 6.92 cases per 100,000 individuals annually, while its prevalence is reported at 60.82 cases per 100,000 individuals [100].

Epidemiological studies have highlighted the impact of geographical location and ethnicity on the prevalence of primary SS, revealing that individuals of non-European descent exhibit over two-fold the prevalence compared to their European counterparts, in addition to demonstrating distinct disease profiles [101]. A cross-sectional analysis from the Big Data Sjögren Project Consortium found that SS is diagnosed an average of seven years earlier in Black or African American patients than in White patients. Furthermore, the highest female-to-male ratio was observed among Asian patients (27:1), while the lowest was noted in Black or African American patients (7:1) [102].

While SS does not correlate with an increased risk of mortality compared to the general population, it significantly impacts patients’ daily activities due to a high incidence of fatigue, depression, anxiety, and reduced physical performance [103].

As no curative therapy for SS currently exists, treatment strategies primarily aim to mitigate the symptoms associated with exocrine gland dysfunction and manage systemic, extra-glandular involvement. In 2019, the EULAR task force issued revised guidelines for the therapeutic management of SS, encompassing both topical and systemic treatment modalities [104]. These guidelines emphasize symptomatic relief rather than defining specific disease activity targets.

The initial management of sicca symptoms should prioritize local therapies, reserving systemic interventions for cases with active systemic manifestations. Treatment of organ-specific involvement should follow a stratified approach, escalating therapy according to disease severity and response. Glucocorticoids should be prescribed at the lowest effective dose for the shortest necessary duration to control disease activity. In cases requiring prolonged immunomodulation, conventional immunosuppressants may serve as glucocorticoid-sparing agents. For patients presenting with severe or treatment-refractory systemic features, B-cell-directed therapies, such as rituximab, epratuzumab, or belimumab, may be considered [104].

### 6.2. Pathogenesis of Fibrosis in SS

Fibrotic alterations have emerged as a relevant pathological component in SS, extending beyond the traditionally recognized exocrine dysfunction to involve multiple organ systems, including salivary glands, lungs, heart, and kidneys. Although SS has been predominantly categorized as an autoimmune disorder targeting exocrine tissues, recent insights indicate that fibrogenesis plays a critical role in disease progression and multi-organ compromise [105].

Within the salivary glands (SGs), persistent inflammatory activity is thought to induce epithelial cell reprogramming via the EMT, contributing to both tissue atrophy and fibrotic remodeling [106]. This process is marked by downregulation of epithelial-specific markers, including E-cadherin and tight junction-associated proteins, consistent with an EMT phenotype (Table 1) [107]. Evidence supports a direct relationship between chronic inflammation, glandular atrophy, and the development of fibrotic lesions, which are associated with impaired secretory capacity, xerostomia, and reduced saliva production [106]. Pulmonary manifestations of SS frequently involve ILD, with additional presentations including airway abnormalities and lymphoproliferative conditions [108,109]. ILD represents the most prevalent and clinically impactful pulmonary complication, affecting approximately one-fifth of SS patients. In some individuals, ILD progresses to a fibrosing phenotype characterized by persistent alveolar damage and excessive extracellular matrix deposition, leading to pulmonary fibrosis. This form of lung involvement is associated with significant morbidity, impaired respiratory function, and increased mortality. A meta-analysis of 23 studies comprising 6157 patients with primary Sjögren’s syndrome (pSS) reported a pooled prevalence of interstitial lung disease (ILD) of approximately 13% (95% CI: 9–19%) [110].

The identification of prognostic indicators and implementation of effective screening strategies, particularly in individuals with idiopathic pulmonary fibrosis (IPF), may facilitate earlier recognition of SS-associated lung disease [111]. However, the utility of universal pulmonary screening in SS remains a topic of debate.

Renal involvement is another recognized extra-glandular manifestation of SS, most commonly presenting as tubulointerstitial nephritis (TIN). This form of nephropathy is characterized histologically by lymphocytic infiltration of the renal interstitium, tubular atrophy, and interstitial fibrosis. These pathological features mirror the glandular infiltration observed in SS and contribute to progressive renal functional decline [112]. Fibrotic changes in the kidney, particularly in patients with chronic or recurrent TIN, can lead to irreversible organ impairment [113,114].

Cardiac fibrosis has also been increasingly identified as part of the systemic involvement in SS. Cardiac magnetic resonance imaging (cMRI) studies have revealed a substantial frequency of myocardial fibrosis, including in patients without clinically apparent cardiac symptoms [115]. The infiltration of lymphocytes into myocardial tissue has been proposed as a central mechanism underlying this fibrosis. Intriguingly, a strong association has been observed between high salivary gland focus scores (FS ≥ 3) and myocardial fibrotic changes, suggesting that cardiac fibrosis may reflect broader systemic disease activity [115]. Non-contrast cMRI has shown promise in detecting early cardiac involvement, thereby offering a valuable, non-invasive diagnostic approach for assessing subclinical cardiac pathology and informing prognosis in SS [116].

Persistent inflammation within the salivary glands in SS is primarily sustained by effector T cell-derived pro-inflammatory cytokines, including IFN-γ, TNF-α, IL-21, and IL-6 [117]. Among these, IFN-γ has been shown to upregulate the chemokine CXCL10, which promotes the recruitment of immune cells by interacting with the CXCR3 receptor expressed on their surface (Table 1) [118,119]. This CXCL10/CXCR3 axis not only facilitates T cell migration but also contributes to the infiltration of B cells into the glandular epithelium, thereby exacerbating the local immune response [120].

The chronic accumulation of immune cells within glandular tissues can drive a pathological progression toward fibrosis. TGF-β, predominantly secreted by regulatory T cells, plays a central role in the fibrotic transformation of glandular tissues by inducing EMT. In patients with primary SS, TGF-β1 mediates fibrogenesis in the salivary glands via the classical TGF-β1/SMAD/Snail signaling cascade [121]. The transcription factor Snail, activated downstream of SMAD2/3 phosphorylation, drives the transcription of EMT-associated genes, promoting phenotypic changes in epithelial cells. In this context, cytokines such as IL-6, IL-17, and IL-22 also converge on the TGF-β pathway and contribute to the fibrotic process through synergistic or parallel mechanisms [122]. Cytotoxic CD8+ T cells exacerbate epithelial damage by inducing the apoptosis of salivary gland epithelial cells (SGECs), a process mediated by direct cell-to-cell contact and the activation of death pathways including Fas/FasL, as well as through cytotoxic molecules such as granzyme B, perforin, IFN-γ, and TNF-α [117]. B cells, upon activation, may differentiate into antibody-secreting plasma cells and support the formation of ectopic germinal centers, often guided by the CXCL10/CXCR3 signaling axis [119]. Macrophages also contribute to fibrotic remodeling by secreting MMPs, which are key enzymes responsible for the breakdown and remodeling of ECM [123]. Furthermore, hypoxia-inducible factor-1 alpha (HIF-1α), a transcription factor regulating cellular responses to hypoxia, has been implicated in TGF-β1-driven fibrogenesis [124]. Experimental models have shown that the deletion of the HIF-1α gene in alveolar macrophages leads to diminished TGF-β1 expression and attenuation of bleomycin-induced pulmonary fibrosis, highlighting a mechanistic link between hypoxia and fibrotic progression [125]. Collectively, these immune cell subsets and signaling pathways establish a pro-fibrotic microenvironment, fostering structural tissue remodeling and contributing to glandular dysfunction in SS.

In the context of fibrotic mechanisms in SS that are independent of immune cell activity, the differentiation of fibroblasts into myofibroblasts represents a critical event in the progression of salivary gland (SG) fibrosis [117]. This cellular transition contributes to ECM deposition, the development of tertiary lymphoid structures (TLS), and the sustained presence of pro-inflammatory cytokines. The dynamic balance between MMPs, particularly MMP-2 and MMP-9, and their endogenous inhibitors, such as TIMP-1 and TIMP-2, regulates ECM remodeling. Disruption of this balance may impair ECM degradation and promote fibronectin accumulation, thereby exacerbating fibrotic changes within the glandular tissue.

### 6.3. Current and Emerging Therapies in SS

A wide range of therapeutic options is currently employed to alleviate sicca symptoms in SS, including artificial saliva and systemic secretagogues such as pilocarpine and cevimeline, which function as muscarinic receptor agonists and have demonstrated efficacy in reducing xerostomia symptoms [126]. Several monoclonal antibodies targeting B-cell-specific markers have shown clinical promise. These include rituximab, directed against CD20; epratuzumab, which targets CD22; and belimumab, an inhibitor of BAFF (Table 2) [127,128,129]. Emerging therapeutic approaches for salivary gland fibrosis increasingly focus on modulating inflammation and promoting tissue regeneration. One strategy involves the use of small interfering RNA (siRNA) to silence ETS1, a transcription factor that regulates MMP-9 expression, leading to a reduction in MMP-mediated ECM degradation (Table 2) [130]. Another innovative approach involves the use of chimeric antigen receptor (CAR) T cells engineered to target fibroblast activation protein (FAP), a molecule highly expressed by fibroblasts with myofibroblast differentiation potential (Table 2). This strategy has been explored in the context of oral submucous fibrosis (OSMF), offering a potential avenue for anti-fibrotic intervention. Among regenerative therapies, MSCs have garnered substantial attention. MSC-based treatments have demonstrated efficacy in modulating the SG microenvironment, suppressing inflammatory responses, and reversing fibrosis by replacing damaged matrix structures [131,132,133,134]. Several preclinical and clinical studies have highlighted the therapeutic impact of MSC administration in SS. In a clinical study by Xu et al., treatment with umbilical cord-derived MSCs led to a decline in serum levels of anti-SSA/Ro and anti-SSB/La antibodies in 24 SS patients [134]. Additionally, MSC therapy has been linked to reduced levels of IL-12, a pro-inflammatory cytokine produced by dendritic cells (DCs), suggesting immunomodulatory effects through the inhibition of DC maturation and cytokine secretion [135,136]. Another investigation involving intravenous MSC infusion in 38 SS patients reported increased IL-27 levels and a reduced Th17/Treg ratio, alongside elevated Treg frequencies in peripheral blood mononuclear cells (PBMCs), reflecting clinical symptom improvement [137]. Despite these benefits, concerns remain regarding MSC differentiation into myofibroblasts, which can exacerbate fibrosis, and the potential for TGF-β1-driven immune dysregulation and excessive ECM deposition [138,139]. Furthermore, chromosomal abnormalities in MSCs have been linked to tumorigenicity [140], and long-term clinical data report notable rates of infection and mortality following MSC therapy in autoimmune conditions [141]. These findings highlight both the promise and the risks associated with MSC-based therapies and emphasize the need for further investigation to ensure safety and efficacy in SG fibrosis.

## 7. Inflammatory Bowel Disease (IBD)

### 7.1. Clinical Presentation and Epidemiology of IBD

IBD is widely recognized as a common chronic immune-mediated disease that occurs primarily in the intestine, and the presence of fibrosis is associated with persistent inflammation. IBD is characterized by the development of fibrotic strictures due to chronic inflammation and dysregulated repair mechanisms. Among IBD, two disease entities predominate: ulcerative colitis (UC) and Crohn’s disease (CD). They are chronic, heterogeneous, lifelong diseases with a young age of onset and high potential for disability. As described by M. Vath, the natural history of inflammatory bowel diseases is influenced by many factors of both environmental and genetic origin [142]. In CD, nearly 30% of patients suffer from fibrosis-related morbidity, while fibrosis is seen to a lesser extent in patients with ulcerative colitis [143].

According to estimates, more than 1 million people in the US and 2.5 million in Europe suffer from IBD [144]. An estimated 2.5–3 million cases of IBD, or about 0.4% of the population, were reported in Europe in 2020. Incidence ranges (per 100,000 person-years) are 0.4–22.8 for CD and 2.4–44.0 for UC [145].

The clinical features of IBD, among chronic or remitting/relapsing inflammatory diseases of the intestinal tract [146], can be divided into gastrointestinal, extraintestinal, and systemic symptoms. Among gastrointestinal features, the typical symptoms like abdominal pain are observed in 62–95% of CD cases and 33–76% of UC cases. Other common disorders include diarrhea (CD: 52–78%; UC: 67–93%) and rectal bleeding (UC: 97%; CD: 14–60%). Urgency and mucous discharge occur mainly in patients with UC. Weight loss and anorexia are more common in CD (weight loss 43–92%) compared to UC (22–55%). Perianal disease occurs in ~25% of patients with CD and is rare in UC [147]. Both diseases are characterized by exacerbations and remissions, and symptoms are often insidious, sometimes preceding diagnosis by years. Extraintestinal manifestations occur in both CD and UC, affecting many systems: musculoskeletal system (arthritis, osteoporosis, osteopenia), skin (erythema nodosum), eyes (epiphoritis, uveitis, scleritis, conjunctivitis), liver and biliary tract (primary sclerosing cholangitis, especially in UC), hepatic steatosis, hematopoietic system (anemia, risk of thrombosis), renal, pulmonary, vascular, endocrine, and neurological systems [147]. Patients with IBD often suffer from systemic and psychological symptoms, such as fatigue and malaise, associated with the active stage of the disease, as well as depression and anxiety, significant symptoms of which have been reported in 65% of women. In pediatric patients, growth retardation was observed in ~30% of CD and ~6% of UC [147].

### 7.2. Pathogenesis of Fibrosis in IBD

Fibrosis in the intestinal tract is an elaborate procedure involving multiple elements and mechanisms, e.g., chronic inflammation, the presence of intestinal mesenchymal cells, and ECM remodeling. In normal conditions, fibrosis occurs due to tissue repair post-injury to protect the architecture of tissues. It is well known that acute inflammation is a key driver of multi-organ fibrosis. In patients with IBD, recurrent and chronic inflammation causes continuous inflammation of the mucosa, leading to the deposition of ECM proteins (e.g., fibronectin, collagen), resulting in progressive fibrosis [148].

The most important elements influencing fibrosis include inflammatory substances from several sources, such as growth factors, as well as different interleukins. It has also been shown that sustained intestinal inflammation leading to high levels of cytokines (e.g., TGF-β, TNF-α, IL-1β, IL-6, IL-17) provokes activation of fibroblasts, converting them into ECM-producing myofibroblasts [149,150] (Table 1). The study of X. Meng et al. confirmed that TGF-β1 stimulates collagen production, increases the conversion of fibroblasts to myofibroblasts, and inhibits matrix breakdown through Smad signaling [151]. In addition, elevated levels of phosphorylated Smad2/3 were observed in the mucosa of CD patients [152]. Another cytokine, IL-17A, is involved in increasing collagen and TIMP-1 production, inhibiting ECM degradation, and is up-regulated in constricted intestinal areas [150,153]. TNF-α has been studied to promote myofibroblast proliferation and the ECM deposition [147]. Moreover, Th2 cytokines (IL-4, IL-13) further enhance collagen formation and TGF-β activation [154] (Table 1). A study of mouse models of bleomycin-induced pulmonary fibrosis and carbon tetrachloride-induced liver fibrosis showed that short-term exposure to the drug induced epithelial cell apoptosis and hepatocyte necrosis [155]. Exposure to the drug led to activation of inflammatory factors and wound healing reactions, which in turn led to ECM deposition in tissues and fibrosis formation [143].

The two processes of EMT and EndoMT have also been confirmed to be important in the formation of fibrosis. It has been shown that damaged epithelial/endothelial cells in IBD can transform into myofibroblasts, thus consolidating fibrosis. The presence of EMT is well documented in Crohn’s strictures [149]. EMT acts as both a wound healing mechanism and a fibrosis inducer, depending on the balance between ECM synthesis and degradation [149,156].

ECM accumulation can be regulated by MMPs and their inhibitors, TIMPs [157]. Among other factors, matrix remodeling takes place with the participation of MMPs and TIMPs, and an imbalance between them is crucial for the occurrence of fibrosis. Myofibroblasts overproduce collagen (types I and III), fibronectin, and TIMPs, while MMP activity is suppressed, leading to ECM accumulation [123]. Recently, S. Li et al., in a bioinformatics study, identified MMP2 and COL1A2 as common markers of fibrosis in IBD and liver disease [158].

The state of the microbiota is also important in the pathogenesis of fibrosis. A recent study by L. Chen et al. confirmed that dysbiosis and altered bacterial signaling through TLRs exacerbate chronic inflammation and fibrosis progression [159]. TLR2 and TLR4 are involved in the induction of inflammation, while TLR2/IL-10 and TLR9 mediate immunosuppression to maintain intestinal homeostasis. In addition, microbiota-induced factors such as TL1A and IL-33 directly stimulate ECM deposition and fibrogenic responses [160].

GWAS have identified several genetic pathways for IBS pathogenesis, but most of them have not been confirmed yet. Genetic susceptibility has been associated with genes, e.g., NOD2, TLR4, IL23R, IL12B, JAK2, CX3CR1, STAT3, ATG16L1, IRGM, FUT2, TGF-β, MMP3, and MAGI1, which modulate inflammatory and fibrotic pathways [161,162]. GWAS have identified autophagy genes relevant to IBD, underlying susceptibility, especially to CD. The mutation of genes in the autophagy pathway (e.g., IRGM, lrrk2, Ulk1, Atg16l1, and Nod2) predisposes individuals to severe CD. Furthermore, impaired autophagy in mesenchymal cells may enhance ECM accumulation, and recent findings underscore its dual role in fibrogenesis [163].

### 7.3. Current and Emerging Therapies in IBD

The etiopathogenesis of IBS is comprehensive, and available treatment strategies and emerging therapies are based on different mechanisms of fibrosis, but they have not been implemented in clinical practice. Surgical interventions are not a common practice in patients with IBD. The mainstay of treatment is lifestyle modification, diet, additional supplementation, and medications to alleviate specific IBD symptoms. Conventional therapy includes anti-inflammatory (aminosalicylates, corticosteroids) and immunosuppressive approaches with immunomodulators (e.g., Thiopurines and Methotrexate), which are often part of combination therapy with advanced therapies (TNF inhibitors, integrin/interleukin antagonists, JAK inhibitors, S1p modulators) [164]. Unfortunately, no drugs have yet been approved to reverse or prevent fibrosis in IBD. However, some early-stage clinical trials are promising. Based on the mechanisms of fibrosis, potential therapeutic targets can be divided into two groups: pro-fibrogenic mediators (e.g., TGF-β1, IL-1, IL-4, IL-6, IL-11, IL-13, IL-17, IL-33, IL-34, IL-36, TL1A, CTGF, IGF-1, cadherin 11, flagellin) (Table 2) and anti-fibrogenic mediators (e.g., IFN-γ, IL-12) [165]. In this paper, we will focus on the most promising therapeutic targets for the treatment of fibrosis.

With regard to pro-fibrogenic mediators, the main player is TGF-β1 with all its downstream effectors. TGF-β1 levels have been shown to be strongly correlated with the activation of angiotensin II, which is observed in the colonic mucosa of CD patients. Based on this, it was hypothesized that angiotensin-converting enzyme (ACE) inhibitors and sartans (angiotensin II receptor antagonists) may play a role in intestinal fibrogenesis (Table 2). The first ACE inhibitor tested was captopril, which proved effective in preventing colonic fibrosis in TNBS-induced colitis in rats [166,167]. The relevance of the peroxisome proliferator-activated receptor γ (PAPPR-γ) as a natural protective agent against fibrosis was confirmed in the context of inflammatory and fibrotic mechanisms in many diseases with fibrosis symptoms. The antifibrotic effect of a novel 5-ASA analog able to activate the PAPPR-γ (GED-0507-34, Levo) was used to treat colonic fibrosis induced in mice (Table 2). It was confirmed that GED treatment reduced the TGF-β and ACTA1 expression in primary human intestinal fibroblasts from UC patients [168]. Recently, Pirfenidone (5-methyl-N-phenyl-2-(1H)-pyridone, PFD) has emerged as a promising anti-fibrotic agent, showing significant efficacy and multifaceted effects. In vivo studies have shown that PFD effectively inhibits the activation of Smad and MAPK pathways associated with TGF-β1 [169].

An extensive group of therapeutic targets in the treatment of fibrosis is metalloproteinases. The presence of the metalloprotease Adamdec1 is essential at the interface between tissue remodeling and healing in colitis [170].

Rho kinase (ROCK) plays multiple roles in TGF-β-induced myofibroblast activation, which may also be therapeutic targets in fibrotic diseases. The use of a ROCK inhibitor (AMA0825) reduced the TGF-β1-induced activation of myocardin-related transcription factor and p38 mitogen-activated protein kinase (MAPK), reducing the secretion of matrix metalloproteinases, collagen, and IL6 from fibroblasts. It is noteworthy that combining AMA0825 with anti-inflammatory agents such as anti-TNF-α in vivo alleviated inflammation but also prevented fibrous tissue accumulation, highlighting the importance of combination therapy [166,171] (Table 2). It is worth highlighting the great potential and importance of microbiome modulating strategies (e.g., Lactococcus lactis-NCDO2118 FnBPA+ in alleviating fibrosis) and the use of natural extracts (e.g., Daikenchuto, Maggot extract, Curcumin, Resveratol) [172] in the treatment of IBD, which are not described in this article.

Despite numerous studies on the mechanism of fibrosis, key cellular factors and biomarkers for the early detection of fibrosis in IBD are still not fully understood. Based on selected data, it seems that a personalized approach and a combination of anti-inflammatory and anti-fibrotic strategies are crucial in the treatment of IBD.

## 8. Rheumatoid Arthritis (RA)

### 8.1. Clinical Presentation and Epidemiology of RA

RA is a disorder that belongs to Systemic Autoimmune Rheumatic Diseases (SARDs), a group of immune-mediated disorders characterized by diverse clinical manifestations. The main causes of SARD development include environmental, genetic, and immune factors, with a strong impact of inflammatory responses. RA affects approximately 0.5 to 2% of the general population. Particularly, women, smokers, and those with a family history of the disease are often affected. In a cross-sectional study with the use of the Global Burden of Disease (GBD), Z. Zang et al. indicated that the RA-related incidence rate increased from 11.66 to 13.48 per 100,000 population from 1990 to 2021 [173]. The initial manifestation of RA is characterized by symmetric polyarticular pain and swelling, and additionally, some nonspecific systemic symptoms, e.g., fatigue, low-grade fever, muscle aches and pains, or weight loss are observed. RA initially affects the small joints, e.g., proximal interphalangeal joints and metacarpophalangeal joints, and further spreads to larger ones. RA is not limited to joint involvement alone, and due to its inflammatory characteristics, it can affect various organs, including the heart, lungs, skin, and eyes, which is observed among approximately 17.8–40.9% of RA patients [174,175].

In the active phases of RA, C-reactive protein (CRP) levels and the erythrocyte sedimentation rate (ESR) are elevated. RA can also be characterized by the presence of autoantibodies, such as RF, which is not specific for RA, unlike anti-citrullinated protein antibody (ACPA), which is considered highly specific for the disease and can be detected long before the first symptoms of RA appear [175]. From a genetic point of view, the study by P. A. Juge et al. confirmed that RA is a complex genetic phenotype, with the minor allele of the mucin 5B-oligomeric mucus/gel-forming (MUC5B) gene promoter variant (rs35705950) as a risk factor for the disease. The presence of the MUC5B variant was observed in 50% of patients with RA disease, with the strongest association with rheumatoid arthritis interstitial lung disease (RA-ILD) (especially in patients with usual interstitial pneumonia—UIP and fibrosing hypersensitivity pneumonitis—HP) [176]. Subsequently, whole exome sequencing studies have identified mutations in the TERT, RTEL1, PARN, and SFTPC genes that may be associated with increased susceptibility to RA-ILD [177]. Of the other genetic factors, a set of alleles from the MHC class II region has been identified as a risk factor for developing RA in up to ~40% of cases. In particular, there are a number of high-risk alleles in HLA DRB1, such as HLADRB1*0404 [178], whose presence has been confirmed in patients with elevated RF or ACPA levels. In addition, a conserved amino acid sequence in the HLA-DRB chain was strongly associated with the presence of antibodies to cyclic citrullinated peptides (anti-CCP) and the development of RA [179].

Chronic inflammation is one of the main factors causing fibrosis, which affects many organs in RA. The presence of chronic inflammation in RA (synovitis) can also lead to fibrosis in the joints themselves, contributing to their stiffness and limited range of motion. In addition, fibrosis can also occur in other organs affected by RA, such as the heart (myocardial fibrosis) and kidneys, although lung involvement is the most pronounced [174,175]. The primary presence of fibrosis in the lungs leads to a serious condition, which is RA-ILD [180]. Clinically significant ILD is observed in 10% of RA patients, while the prevalence of subclinical abnormalities consistent with ILD has been documented as up to 60–80% patients [175,181]. Fibrosis in RA-ILD is characterized by lung distortion with traction bronchial dilatation and/or honeycombing. The main risk factors for fibrosis progression in RA-ILD are diabetes mellitus co-occurrence, elevated Disease Activity Score in 28 joints-Erythrocyte Sedimentation Rate (DAS28-ESR) levels, and advanced high-resolution computed tomography scores [174]. Although synovitis is a key feature of RA, recent reports indicate that initial autoimmunity can develop in tissues outside the joints, particularly the lungs [182]. It is believed that in genetically predisposed individuals and under the influence of environmental factors such as smoking, an initial violation of immune tolerance and inflammation may occur.

### 8.2. Pathogenesis of Fibrosis in RA

The mechanisms driving fibrosis in RA are mainly connected with chronic inflammation in joints (synovitis) and fibrotic transitions in lung tissue. The mechanisms that occurred are comprehensive and are mainly connected with synovitis, activation of fibroblasts, and their differentiation into myofibroblasts and deposition of ECM.

One of the major players among the mechanisms of fibrosis in both joints and lungs in RA is TGF-β1/3. It has been shown that RA synovial fluid is rich in active TGF-β and activates canonical Smad2 signaling, inducing α-SMA expression in MSCs and fibroblasts. It was also confirmed that RA synovial fluid induces α-SMA via TGF-β1/Smad2, while blocking TGF-βRI or neutralizing TGF-β prevents myofibroblast conversion, confirming the key role of this pathway [183]. Recently, the study of K. Bhamidipati et al. on biopsies from RA patients has identified an expansion of fibrogenic fibroblasts characterized by the high expression of the fibrosis-associated extracellular matrix protein COMP. COMP fibroblasts were localized in perivascular niches and were significant for TGF-β activity. Notch signaling was shown to be an upstream regulator of fibroblast TGF-β signaling [184]. Moreover, disruption of Notch signaling in vitro enabled activation of fibrogenic fibroblasts. Other studies have found that sputum-derived TGF-β levels were significantly elevated in patients with RA and pulmonary fibrosis, which correlated with more severe lung disease. Moreover, TGF-β levels were higher in patients with the MUC5B polymorphism associated with ILD risk and were also associated with a higher probability of RA and pulmonary fibrosis, even after accounting for age and gender [185]. Based on the above studies, TGF-β inhibition can be considered as a potential therapeutic for anti-fibrotic therapy.

Under normal conditions, fibroblast-like synoviocytes (FLSs) are crucial for maintaining cartilage integrity and function by producing ECM and synovial fluid components. In the context of RA, these cells become hyperactive and hyperproliferative with an aggressive phenotype that alters the joint microenvironment by producing MMPs that degrade the ECM. It is known that in RA, synovial fibroblasts can promote inflammation and cartilage destruction, while the interaction between T cells and synovial fibroblasts promotes the activation of synovial fibroblasts [182]. Inflammatory cells produce TNF-α, which induces vascular endothelial cells and synovial membrane cells to produce CXCL13, which recruits circulating B and T cells to the site of inflammation [186,187] (Table 1). Recently, many reports have focused on studies using single-cell RNA sequencing (scRNA-seq) technology to investigate the interaction of synovial fibroblasts and macrophages, which may be relevant to the mechanism of RA development. Pathogenic fibroblasts, such as DKK3-SLF and CD34-SLF, have been shown to produce excessive amounts of cytokines and chemokines (IL-6, CCL2), which can cause M1 polarization of macrophages and increased secretion of TGF-β, IL-6, and PDGF [188], which are pro-fibrotic cytokines. These products enhance the inflammatory response of both macrophages and fibroblasts, leading to fibrosis and hypertrophy of the synovial membrane.

The EMT process is also relevant to the occurrence of fibrosis in RA. The EMT process of the synovial membrane in RA joints has been observed. It was shown that synovial lining cells in healthy tissue expressed epithelial markers (e.g., E-cadherin, collagen IV), while samples from RA patients expressed the myofibroblast marker α-SMA (Table 1). Moreover, it was also shown that healthy FLS stimulated with RA synovial fluid or TGF-β increased mesenchymal markers, e.g., α-SMA, collagen I [189]. RA-FLS have been confirmed to undergo TGF-β1-mediated EMT, with decreased E-cadherin and increased N-cadherin, vimentin, and α-SMA through the Smad2/3 pathway [190]. Additionally, recent reports indicated the lysosome-associated membrane protein 3 (LAMP3) as a mediator of EMT in RA-FLS, linking synovial cell invasiveness and fibrosis to epithelial transition [191].

Fibrosis of the synovial membrane is known to lead to the accumulation of ECM proteins in RA patients, which is relevant to the clinical manifestations of the disease. A study of circulating fibrocytes, potential precursors of FLS, showed that fibronectin and MMP levels were higher in FLS compared to fibrocytes. RA-derived fibrocytes (circulating precursors) were compared with synovial fluid and FLS tissues and found that fibronectin and MMP3 were higher in FLS, while fibrocytes expressed more MMP9 [192]. These results showed that fibrocytes express ECM molecules and cytokine receptors and highlighted the different ECM and MMP profiles among stromal cell types, which may be due to their different functions or differentiation status during RA. S. Madsen et al. examined TGF-β/SMAD and BMP signaling pathways in RA and showed that they present a “fibroid phenotype” with up-regulated TGF-β, SMAD, and BMP pathways. In vitro model studies from primary FLS from joints showed that TGF-β induced a fibrotic response, which was characterized by increased levels of ECM proteins such as fibronectin and collagen types I, III, and VI [193]. RA-ILD involves dysregulated ECM turnover mediated by MMPs/TIMPs, myofibroblast transformation, epithelial damage, and persistent collagen deposition. The majority of fibrogenic pathways are common to synovial and pulmonary fibrosis, including TGF-β/SMAD signaling and type I/III collagen dysregulation [194]. In vitro studies on human type II alveolar cells have shown that the EMT can be induced by treatment with agents such as TGF-β and IL-6 and has been reported to be inhibited by blocking the JAK/STAT signaling pathway. IL-6-induced EMT processes in alveolar epithelial cells were shown to be modulated by MTX or JAK inhibitors, affecting fibrosis progression [195,196]. The key mechanisms of fibrosis development are complex and not fully elucidated. Further research is needed to fully understand these mechanisms in order to improve RA treatment strategies.

### 8.3. Current and Emerging Therapies in RA

RA treatment focuses mainly on reducing inflammatory processes and alleviating symptoms of the disease. It was shown that PFD could effectively inhibit fibroblast proliferation, inhibit local inflammatory cell infiltration, reduce collagen deposition, and, furthermore, regulate wound healing and inhibit endothelial cell angiogenesis [197]. In addition, PFD is used as an anti-fibrotic agent in idiopathic pulmonary fibrosis treatment, which shares many clinical manifestations with RA-ILD [198]. Taken together, it was assumed that PFD may be potentially used in RA treatment. PFD was tested in a collagen-induced arthritis model in rats and significantly relieved joint swelling, synovial hyperplasia, inflammatory cell infiltration, and joint destruction. PFD application was also associated with reduced expression of pro-inflammatory, chondrogenic, and angiogenic cytokines (IL-1β, IL-6, IL-8, MMP-1/3/2/9, and VEGF) (Table 2). It was also shown that PFD alleviates the local inflammatory microenvironment by inhibiting the STAT3 and AKT/NF-κB signaling pathways [197]. These findings suggest that PFD may inhibit inflammation and angiogenesis through multiple pathways and serve as a potential therapeutic drug for RA. These findings suggest that PFD can inhibit inflammation and angiogenesis through multiple pathways, making it a potential therapeutic drug in RA.

Other drugs being studied for the treatment of RA include nintedanib, commonly used in pulmonary fibrosis, and tofacitinib, a JAK inhibitor [199]. These drugs have been shown to inhibit the TGF-β-induced formation of collagen I/III/VI and fibronectin in joint FLS [193]. This indicates that both agents may have direct anti-fibrotic effects on synovial tissue, in addition to their known systemic anti-inflammatory or anti-fibrotic effects in the lungs.

MSC-based therapeutic approaches have gained significant attention in RA treatment. Preclinical and early-phase clinical studies using bone marrow or umbilical-cord MSCs showed upregulation of TGF-β1 and IL-10, suppression of NF-κB activity in synovial fibroblasts, and reduced fibroblast cadherin-11 expression and invasive, inflammatory behavior [200]. A new drug repositioning method has been used by Z. Wei et al. to prepare drug screens [187]. Based on the established differentially expressed genes (DEG) database, 16 fibrosis-related drugs have been successfully repositioned in RA (e.g., emodin, HDAC inhibitors, silymarin, auranofin, penicillamine, azathioprine, sulfhydryl compounds, isoxazoles) for future validation. In conclusion, preclinical models confirm the direct anti-fibrotic effect of nintedanib, tofacitinib, and pirfenidone in the treatment of RA (Table 2). For RA-ILD, antifibrotic agents represent the best supported therapy to date.

## 9. Fibrosis Regression

Anti-fibrotic drugs, for instance, nintedanib and pirfenidone, aimed at halting fibrotic progression, do not necessarily restore organ functionality, as the fibrotic ECM often retains biomechanical and biochemical cues that perpetuate the activation of myofibroblasts. Therefore, functional recovery necessitates the selective removal or replacement of this pathological ECM with a structurally and functionally normal matrix in a regulated manner [201]. Accumulating evidence from both preclinical models and clinical observations indicates that fibrosis can regress, particularly within the hepatic environment [202]. While end-stage cirrhosis remains largely refractory to current therapies, patients with early-to-intermediate cirrhosis caused by hepatitis C virus infection often exhibit spontaneous fibrosis regression following antiviral treatment [203]. This reversal is strongly associated with ECM remodeling and degradation [204].

It was indicated that organs with limited regenerative potential, such as the lungs, exhibit a capacity for fibrosis resolution [201]. In cases of acute respiratory distress syndrome, substantial resolution of pulmonary fibrosis has been documented [205]. Although animal studies have demonstrated that fibrotic lesions in the lung can resolve following cessation of fibrogenic insults, such as bleomycin, asbestos, or fluorescein isothiocyanate, chronic fibrosing conditions in humans, including IPF and SSc-ILD, typically exhibit a progressive and treatment-resistant phenotype. However, randomized clinical trials in patients with SSc-ILD have identified subsets of individuals who exhibit measurable improvements in pulmonary function, suggesting the potential for tissue regeneration or fibrosis reversal under specific therapeutic conditions [206]. Cutaneous fibrosis also demonstrates plasticity. Clinical data suggest that skin fibrosis in SSc patients may regress spontaneously or following therapeutic intervention. The retrospective cohort study identified spontaneous regression of skin fibrosis in 11% of Thai patients with SSc [207]. The authors propose that the relatively high frequency of skin regression observed in this population may be attributable to a milder clinical phenotype commonly seen among Thai SSc patients, potentially reflecting underlying genetic or ethnogeographic factors in comparison to Western populations [207].

The propensity for fibrotic regression appears to be organ-dependent and may vary according to the etiology of the fibrogenic insult and host-specific variables such as genetic predisposition, age, sex, and immune status [208].

The resolution of fibrosis is primarily driven by the degradation of aberrant ECM components and the restoration of the matrix’s physiological mechanical and biochemical properties. In experimental models, targeted induction of myofibroblast apoptosis has been shown to trigger fibrosis resolution. Nonetheless, the precise cellular and molecular pathways governing ECM degradation post-myofibroblast clearance remain inadequately defined.

Various cell types, including macrophages, fibroblasts, natural killer (NK) cells, and neutrophils, have been implicated in ECM degradation through their production of MMPs [3].

The dismantling of fibrotic ECM, primarily composed of heavily cross-linked type I collagen, is initiated by collagenases such as MMP-1, MMP-2, MMP-8, MMP-13, MMP-14, MMP-15, and MMP-16. Subsequent degradation is mediated by gelatinases (MMP-2 and MMP-9) and other proteolytic enzymes like cathepsin K [209]. Following enzymatic degradation, collagen fragments are internalized by various cell types, including fibroblasts and macrophages. In fibroblasts, the uptake of collagen degradation products occurs via the C-type mannose receptor 2, which mediates their trafficking to lysosomes through a non-phagocytic pathway [210]. In addition, fibroblasts are capable of phagocytosing larger collagen fibrils via integrins such as α1β1, α2β1, and α3β1, which similarly target the internalized material to lysosomal compartments for further degradation [211].

## 10. Conclusions

Fibrosis represents a central pathological process in a wide spectrum of autoimmune diseases, contributing significantly to chronic tissue damage and functional decline. As highlighted in this review, the complexity of fibrotic mechanisms, spanning diverse cellular pathways and molecular mediators, underscores the need for a deeper understanding of its role in autoimmune pathology. Given the substantial morbidity and mortality associated with fibrotic complications across multiple organ systems, the development of effective, targeted antifibrotic therapies remains an urgent and unmet clinical need. Continued research into the mechanisms of fibrosis and the translation of this knowledge into innovative therapeutic approaches will be critical for improving outcomes in patients affected by autoimmune-mediated fibrotic disease.

## Figures and Tables

**Table 1:** The role of fibroblasts, cells and cytokines involved in fibrosis in autoimmune and immune-mediated diseases.

Disease	Role of Fibroblasts/Myofibroblasts	Other Key Involved Cells	Main Profibrotic Cytokines
Systemic sclerosis (SSc)	Activated fibroblasts differentiate into myofibroblasts, producing excessive collagen and ECM → progressive skin and organ fibrosis	Th2/Th17 T cells, B cells, M2 macrophages, pDCs, endothelial cells (EndoMT)	TGF-β, IL-6, IL-4, IL-13, PDGF, Endothelin-1, BAFF, IFN-α
Morphea	Local skin fibroblasts become activated, producing collagen and ECM → sclerotic plaques	CD4+ T cells (Th1/Th17→Th2), macrophages, dendritic cells, endothelial cells	TGF-β, IL-4, IL-6, IL-13, IL-17, CTGF, PDGF, INF-γ
Autoimmune hepatitis (AIH)	Hepatic stellate cells (fibroblast-like) transform into myofibroblasts → collagen I/III production → liver fibrosis/cirrhosis	T cells, NK cells, Kupffer cells, dendritic cells, liver sinusoidal endothelial cells (LSECs)	TGF-β, PDGF, TNF-α, IL-6, IL-1β, IL-17, TIMP-1
Systemic lupus erythematosus (SLE)	Myofibroblasts undergo activation → ECM accumulation → fibrotic remodeling in kidneys, lungs, skin, and heart	NET-forming neutrophils, macrophages	TGF-β, IL-6, IL-1β, TNF-α, IFN-α, PDGF
Sjögren’s syndrome (SS)	Epithelial cell reprogramming via EMT, differentiation of fibroblasts into myofibroblasts → ECM accumulation → salivary gland fibrosis	Th1/Th17/Th22, CD8+ T cells, B cells, macrophages	TGF-β1, IFN-γ, TNF-α, IL-6, IL-17, IL-21, CXCL10
Inflammatory bowel disease (IBD)	Intestinal fibroblasts/mesenchymal cells activate into myofibroblasts → collagen deposition → strictures (mainly CD)	Th1/17 T cells, macrophages, epithelial cells (EMT)	TGF-β, TNF-α, IL-6, IL-1β, IL-13/IL-17, TL1A, CTGF, ROCK, microbiota induced TL1A and IL33
Rheumatoid arthritis (RA)	Fibroblast-like synoviocytes (FLS) become aggressive myofibroblast-like cells → synovial and pulmonary fibrosis	Th1/Th17 T cells, macrophages, B cells, neutrophils, endothelial cells, synovial lining cells, circulating fibrocytes	TGF-β1/3, IL-6, TNF-α, IL-17, PDGF, Notch, JAK/STAT, BMP, MMP/TIMP

**Table 2:** Summary of profibrotic mediators, therapeutic inhibition, and antifibrotic development across diseases.

Autoimmune Disease	Key Profibrotic Mediators (Cytokines/Pathways)	Advantages of Inhibition	Potential Drawbacks of Inhibition	Status of Antifibrotic Therapeutic Development
Systemic sclerosis (SSc)	TGF-β, IL-6, IL-4/IL-13, PDGF, CTGF, endothelin-1 (ETaR), AT1R, BAFF, IFN-α/ TGF-β/SMAD pathway, JAK/STAT pathway, EndoMT, TLR-signaling	Inhibition of fibroblast activation and excessive ECM production → slows disease progression, preserves organ function (especially lung), reduces fibrosis-related complications, may improve survival	Immunosuppression leading to infections; treatment-related adverse effects (e.g., diarrhea with nintedanib, hematologic toxicity with HSCT); limited ability to reverse established fibrosis; heterogeneous patient response; off-target effects	Approved: nintedanib (FDA/EMA for SSc-ILD); investigational/repurposed: pirfenidone (not approved in SSc-ILD), biologics: rituximab (anti-CD20), tocilizumab (anti-IL-6), fresolimumab (anti-TGF-β), romilkimab (anti-IL-4/IL-13), abatacept (CTLA-4-Ig), belimumab (BAFF inhibitor); advanced immunotherapies: HSCT; experimental: CD19 CAR-T cells; JAK inhibitors
Morphea (localized scleroderma)	TGF-β, IL-4, IL13, IL-6, PDGF, CTGF, IFN-γ, IL-17, vascular adhesion molecules (E-selectin, VCAM-1)/ TGF-β/SMAD pathway, PDGF/c-Abl pathway, JAK/STAT signalling	Blocking profibrotic pathways may reduce fibroblast activation and collagen deposition, control inflammation, halt disease progression, prevent tissue atrophy and deformities, improve skin elasticity and quality of life	Risk of systemic immunosuppression (infection, toxicity), especially in children; limited ability to reverse established fibrosis; heterogeneity of clinical response; relapse after therapy withdrawal; long-term safety concerns	Current: methotrexate ± systemic steroids (first-line), MMF, cyclosporine, hydroxychloroquine, azathioprine, retinoids; topical steroids/tacrolimus; phototherapy, biologics (rituximab; infliximab); emerging: tocilizumab (anti-IL6), abatacept (a CTLA-4-Ig fusion protein), JAK inhibitors (tofacitinib, baricitinib), imatinib (PDGF/c-Abl), anti-TGF-β, BET/HDAC inhibitors—mainly under early clinical investigation
Autoimmune hepatitis (AIH)	TGF-β, PDGF, TNF-α, IL-1β, IL-6, IL-17, chemokines (CXCL10), TIMP-1/ TGF-β/SMAD pathway, TLR4/TLR9, NLRP3 inflammasome, Wnt/β-catenin signaling	Suppression of inflammation and HSC activation → inhibits collagen deposition, slows progression to fibrosis/cirrhosis, preserves liver function, lowers portal hypertension risk, may reduce need for transplantation	General immunosuppression → infection risk; steroid/azathioprine toxicity; incomplete response or intolerance in some patients; relapse upon dose reduction; difficulty reversing established fibrosis; off-target effects	Current standard: Prednisolone ± azathioprine; budesonide; MMF; calcineurin (cyclosporine A, tacrolimus), mTOR (everolimus), biologics (rituximab, infliximab) for refractory AIH. Emerging: hematopoietic/mesenchymal stem cell therapy; Experimental immune-targeted: Zetomipzomib (immunoproteasome inhibitor), Ianalumab (anti-BAFF-R), JKB-122 (TLR-4 antagonist), JAK-inhibitors (case reports), adoptive Treg transfer, low-dose IL-2
Systemic lupus erythematosus (SLE)	TGF-β/Smad pathway, IL-6, IL-1β, TNF-α, IFN-α (type I interferon axis), PDGF, Wnt/β-catenin signaling, epithelial-to-mesenchymal transition (EMT), NETosis (NET-induced EMT), M2 macrophages, myofibroblast activation	Reduces progression toward organ fibrosis (kidneys, lungs, skin), preserves organ function, improves long-term outcomes, prevents irreversible damage, may decrease morbidity associated with lupus nephritis and interstitial lung disease	Risk of immunosuppression and infection; potential off-target effects; organ-specific variability and heterogeneity of fibrosis; difficulty reversing already established scarring; possibly poor tolerability or efficacy in some patients	Current: immunosuppressants (steroids, MMF, CYC), biologics (belimumab—anti-BAFF; anifrolumab—anti-IFNAR1). Antifibrotics under investigation: nintedanib (tyrosine-kinase inhibitor), anti-TGF-β agents (fresolimumab), EMT/Wnt inhibitors, stem-cell-based therapies (MSC), NET-targeting strategies, TGF-β antisense oligonucleotides; none yet specifically approved for SLE-related fibrosis
Sjögren’s Syndrome (SS)	TGF-β/SMAD/Snail signaling, IFN-γ, TNF-α, IL-6, IL-21, IL-17, CXCL10/CXCR3 axis, EMT of epithelial cells, HIF-1α, MMP/TIMP imbalance, myofibroblast activation, persistent T and B cell–mediated inflammation	Reduces progression of glandular and extraglandular fibrosis (salivary glands, lungs, kidneys, myocardium), preserves secretory and organ function, delays irreversible atrophy and structural remodeling	Immunosuppression risks (infections), difficulty reversing advanced fibrosis, heterogeneous organ involvement, potential off-target effects, possible promotion of additional immune dysregulation	Current: symptomatic (artificial saliva, pilocarpine); immunomodulators (glucocorticoids, hydroxychloroquine, MTX); biologics (rituximab, belimumab, epratuzumab) for systemic disease. Emerging: MSC therapy, siRNAs (e.g., ETS1), anti-TGF-β strategies, FAP-targeted CAR-T cells, anti-fibrotic small molecules—largely experimental or preclinical
Inflammatory Bowel Disease (IBD)	TGF-β/Smad, TNF-α, IL-1β, IL-6, IL-17, IL-13, IL-33, TL1A; EMT/EndoMT; myofibroblast activation; MMP/TIMP imbalance; microbial TLR signaling; autophagy-related pathways; ROCK activation; CTGF; angiotensin II; microbiota-derived factors	Prevents fibrostenotic complications and strictures, limits need for surgical resections, preserves intestinal architecture, improves long-term outcomes and quality of life by limiting fibrosis-driven morbidity	Risk of impairing normal mucosal healing; immunosuppression-related infections; heterogeneity of disease sites and fibrosis course; limited ability to reverse established strictures; potential off-target effects	Current: Anti-inflammatory therapy (5-ASA, steroids, thiopurines, MTX, biologics—anti-TNF, anti-integrin, anti-IL, JAK inhibitors, S1P modulators); no approved anti-fibrotic drugs yet. Emerging candidates: ACE inhibitors/sartans; PPAR-γ agonists (GED-0507-34); pirfenidone; ROCK inhibitors (AMA0825); MMP modulators; microbiome therapies; natural compounds (curcumin, resveratrol); combination anti-inflammatory + antifibrotic approaches under investigation
Rheumatoid arthritis (RA)	TGF-β (Smad2/3), IL-6, TNF-α, IL-17, PDGF, Notch signaling, EMT/EndoMT, synovial fibroblast activation (FLS → myofibroblasts), MMP/TIMP imbalance, JAK/STAT pathway, BMP signaling, COMP-positive fibrogenic fibroblasts	Limits synovial and pulmonary fibrotic remodeling, preserves joint mobility and lung function, reduces risk of irreversible organ damage (i.e., RA-ILD progression), may complement anti-inflammatory therapy to improve prognosis	Possible interference with physiological repair processes; systemic immunosuppression with infection risk; limited reversal of established fibrosis; heterogeneity in fibrotic phenotypes; could impact cartilage homeostasis/angiogenesis	Current RA therapy: Disease-Modifying Anti-Rheumatic Drugs (DMARDs): MTX, sulfasalazine, biologics (anti-TNF, IL-6 inhibitors, abatacept, rituximab) and JAK inhibitors (tofacitinib). Emerging antifibrotics: pirfenidone (preclinical/clinical for RA and RA-ILD), nintedanib (approved for RA-ILD), tofacitinib and other JAK inhibitors show anti-fibrotic effects, MSC-based therapies in early studies; repositioned drugs (HDAC inhibitors, emodin, silymarin) under investigation

## Abbreviations

The following abbreviations are used in this manuscript:

ACPA	anti-citrullinated protein antibody
AIH	Autoimmune hepatitis
ANA	anti-nuclear antibodies
APC	antigen-presenting cells
ASCs	adult stem cells
BAAF	B cell activating factor
CCP	cyclic citrullinated peptides
CD	Crohn’s disease
CI	confidence interval
CKD	chronic kidney disease
Cmri	cardiac magnetic resonance imaging
CRP	C-reactive protein
CXCL	C-X-C motif chemokine ligand
CYC	cyclophosphamide
DAMPs	damage-associated molecular patterns
DAS28-ESR	Disease Activity Score in 28 joints-Erythrocyte Sedimentation Rate
DEG	differentially expressed gene
EC	endothelial cell
ECM	extracellular matrix
DMARDs	Disease-Modifying Anti-Rheumatic Drugs
EMT	epithelial-mesenchymal transition
EndoMT	endothelial-mesenchymal transition
ESR	erythrocyte sedimentation rate
ESRD	end-stage renal disease
FLS	fibroblast-like synoviocytes
GBD	Global Burden of Disease
GERD	Gastroesophageal reflux disease)
GWAS	Genome-wide association studies
HSCT	hematopoietic stem cell transplantation
HSCs	Hepatic stellate cells
IBD	Inflammatory Bowel Disease
ILD	Interstitial lung disease
INF	interferon
HLA	human leukocyte antigen
HP	fibrosing hypersensitivity pneumonitis
LAMP3	lysosome-associated membrane protein 3
LN	lupus nephritis
LPS	lipopolysaccharides
LSECs	liver sinusoidal endothelial cells
MHC	major histocompatibility complex
MMF	mycophenolate mofetil
MMPs	matrix metalloproteinases
MSCs	mesenchymal stem cells
MUC5B	mucin 5B, oligomeric mucus/gel-forming gene
NET	neutrophil extracellular trap
NSIP	nonspecific interstitial pneumonia
PAMPS	pathogen-associated molecular patterns
PAH	Pulmonary arterial hypertension
pDCs	plasmacytoid dendritic cells
PFD	pirfenidone
PDGF	platelet-derived growth factor
RA	Rheumatoid arthritis
RA-ILD	rheumatoid arthritis interstitial lung disease
RF	rheumatoid factor
RTX	Rituximab
SARDs	Systemic Autoimmune Rheumatic Diseases
scRNA-seq	single-cell RNA sequencing
SLE	Systemic lupus erythematosus
SSc	Systemic sclerosis
SRC	Scleroderma renal crisis
TCZ	Tocilizumab
Tfh	T follicular helper cells
TGF	transforming growth factor
TIMPs	tissue inhibitors of metalloproteinases
TLRs	Toll-like receptors
TNF	tumor necrosis factor
UC	ulcerative colitis
UIP	usual interstitial pneumonia
